# Rural Cancer Survivors' Perceived Delays in Seeking Medical Attention, Diagnosis and Treatment: Findings From a Large Qualitative Study

**DOI:** 10.1002/cam4.71036

**Published:** 2025-07-21

**Authors:** Alyssa Taglieri‐Sclocchi, Ingrid Bindicsova, Susannah K. Ayre, Michael Ireland, Sonja March, Fiona Crawford‐Williams, Suzanne Chambers, Jeff Dunn, Belinda C. Goodwin, Elizabeth A. Johnston

**Affiliations:** ^1^ Viertel Cancer Research Centre Cancer Council Queensland Brisbane Queensland Australia; ^2^ School of Psychology University of Queensland St Lucia Queensland Australia; ^3^ School of Exercise and Nutrition Sciences Queensland University of Technology Kelvin Grove Queensland Australia; ^4^ School of Psychology and Wellbeing University of Southern Queensland Ipswich Queensland Australia; ^5^ Centre for Health Research University of Southern Queensland Springfield Queensland Australia; ^6^ Manna Institute University of Southern Queensland Springfield Queensland Australia; ^7^ Caring Futures Institute Flinders University Bedford Park South Australia Australia; ^8^ McGrath Foundation Sydney New South Wales Australia; ^9^ Faculty of Health Sciences Australian Catholic University Banyo Queensland Australia; ^10^ Menzies Health Institute Queensland Griffith University Mt Gravatt Queensland Australia; ^11^ Exercise Medicine Research Institute Edith Cowan University Joondalup Western Australia Australia; ^12^ St Vincent's Health Network Sydney Sydney New South Wales Australia; ^13^ Prostate Cancer Foundation of Australia Sydney New South Wales Australia; ^14^ School of Population and Global Health University of Melbourne Melbourne Victoria Australia; ^15^ Population Health Program QIMR Berghofer Medical Research Institute Herston Queensland Australia

**Keywords:** access to healthcare, cancer survivors, health services, health systems, healthcare disparities, regional and remote

## Abstract

**Aims:**

To investigate rural cancer survivors' self‐reported reasons for perceived delays in initial cancer detection and treatment.

**Methods:**

Within a cohort study, adult cancer survivors who had travelled > 50 km for cancer care, staying at subsidised accommodation lodges in city centres in Queensland, Australia, were invited to complete a structured interview on perceived delays in: (i) seeking medical attention, (ii) receiving their diagnosis and (iii) commencing treatment. Content analysis was used to map self‐reported reasons for perceived delays at each step, which were then categorised based on the perceived source: (i) personal, (ii) healthcare professional, (iii) healthcare system or (iv) other. The self‐reported reasons and perceived sources were summarised using descriptive statistics.

**Results:**

Six hundred and eighty‐six rural cancer survivors completed the interview (18% breast, 15% head and neck, 12% prostate and 12% skin cancer). Almost half (*n* = 320, 47%) of participants perceived a delay at one or more steps. Delays in seeking medical attention were perceived by 132 (19%) participants, mostly related to personal factors (*n* = 67, 51%), including misinterpreting (*n* = 19, 28%) signs and symptoms. Delays in diagnosis were perceived by 161 (23%) participants, mostly related to healthcare professional factors (*n* = 86, 53%), including requiring further opinions or testing for diagnosis (*n* = 30, 35%). Delays in commencing treatment were perceived by 157 (23%) participants, mostly due to healthcare system factors (*n* = 57, 37%), including long waitlists (*n* = 39, 68%). Of the participants who perceived a delay in commencing treatment, comparison with timeframes recommended in the relevant Optimal Care Pathway identified that 57% of perceived delays were actual delays.

**Conclusions:**

Perceived delays in the pathway to initial cancer detection and treatment are common among rural cancer survivors. Improvements in patient–clinician communication could reduce perceived delays, particularly in diagnosis and treatment. Promoting early help‐seeking, participation in cancer screening and improving access to diagnostic and treatment infrastructure may also improve care experiences.

## 1 Introduction

For nearly all cancer types, timely detection, diagnosis and treatment are imperative for increasing the likelihood of survival, with delays along this pathway associated with poorer overall outcomes [1, 2]. However, cancer diagnosis and treatment can be complex and multifaceted, involving multiple appointments, healthcare professionals and services, including community‐based primary care, imaging and treatment centres and specialist reviews [3]. These complexities, combined with the personal circumstances of each individual, can introduce multiple potential delays in receiving timely cancer care [4].

For the almost one‐third of Australians living in rural areas (i.e., outside of a major city) [5], there are additional challenges in accessing cancer care due to the limited availability of local healthcare services, costs incurred from travel and difficulties taking time off work, particularly for small, family‐operated businesses [6, 7]. Previous research indicates that people living with cancer in rural areas of Australia experience additional delays in diagnosis and treatment compared to their urban counterparts [8, 9, 10]. These delays may be contributing to poorer cancer‐related outcomes in this population group. In a narrative review of international studies, cancer survivors who had to travel > 50 miles (i.e., > 80 km) for treatment tended to have a more advanced stage of cancer at diagnosis, lower adherence to treatment protocols, worse prognosis and poorer quality of life [11]. Further, there is substantial evidence that geographical remoteness generally impacts cancer survival, with lower 5‐year survival rates for all cancer types combined among rural cancer survivors compared to their urban counterparts [12, 13, 14, 15, 16].

To date, few studies have investigated rural cancer survivors' experiences of care, such as self‐reported reasons for perceived delays in the pathway to initial cancer detection and treatment, from seeking medical attention to receiving the diagnosis, and then commencing treatment. Small qualitative studies among rural cancer survivors in Australia have identified various factors contributing to delays in diagnosis, such as vague symptoms, work or family commitments, false‐negative test results, long wait times for appointments with general practitioners (GPs), delayed referrals to specialists and issues accessing diagnostic testing facilities [10, 17]. While some of these factors have also been reported by cancer survivors living in urban areas [10], it appears long wait times for appointments with GPs, delayed referrals to specialists and issues accessing diagnostic testing facilities are more common or unique to cancer survivors living in rural areas [10]. Little is known, however, about rural cancer survivors' perceptions of delays across the pathway to initial cancer detection and treatment. Thus, investigating the perceptions of delays from the perspective of the rural cancer survivor can contribute to a better understanding of where and why delays are perceived throughout cancer care and where interventions may need to be directed.

Using qualitative data from a large cohort of rural cancer survivors in Queensland, Australia, this study explores rural cancer survivors' perceptions of delays in seeking medical attention, receiving their diagnosis and commencing treatment, and explores the self‐reported reasons for these perceived delays. Findings can be used to identify areas for intervention to reduce delays in initial cancer detection and treatment for rural cancer survivors. Addressing the underlying causes of perceived delays could contribute to improving patient care experiences and addressing inequities in cancer outcomes for rural cancer survivors.

## 2 Methods

### 2.1 Participants and Recruitment

This analysis used data collected in the Travelling for Treatment study [18], a longitudinal cohort study of rural cancer survivors and their caregivers, tracking patient‐reported outcome measures over time (up to 5 years post‐recruitment). The Travelling for Treatment study team comprised early‐career to senior researchers with backgrounds in psycho‐oncology, public health and cancer survivorship. Methods for participant recruitment and data collection have previously been described in detail [7], with further information outlined elsewhere [18, 19, 20, 21]. Briefly, between September 2017 and June 2020, consecutive adults who had a cancer diagnosis or were undergoing tests for cancer, staying at one of Cancer Council Queensland's (CCQ) subsidised accommodation lodges, were invited to participate in the study. To be eligible to stay at one of CCQ's lodges, guests must be required to travel > 50 km (i.e., > 31 miles) to receive cancer treatment or follow‐up care in Queensland, Australia. Where possible, study invitation packs were distributed by lodge staff to guests during their stay. For guests who checked in after hours, an invitation pack was sent via mail to their home address. One week post‐distribution, a research volunteer contacted guests to follow up on the study invitation and respond to any questions. Ethical approval was obtained from a recognised institutional Human Research Ethics Committee (H17REA152). This analysis is reported according to the Consolidated Criteria for Reporting Qualitative Research (COREQ) (Table S1) [22].

### 2.2 Data Collection

Over the 4‐year study recruitment period, participants completed a one‐on‐one structured interview (approx. 45–60 min) either during their stay at the lodge or via phone, which was neither audio nor visually recorded. Participants and interviewers had no prior relationship. The interviews were conducted by a team of 44 trained interviewers. To ensure consistency and reliability in the data collection across the large team, all interviewers completed mandatory, study‐specific training that covered core interviewing skills, ethical considerations and protocols for managing participant distress. Standardised interview guides and ongoing supervision were used to support uniformity in the conduct of interviews. Participants also completed a written, self‐administered questionnaire that collected information on sociodemographic and clinical characteristics. This analysis reports on qualitative information collected in the interview about participants' self‐reported reasons for perceived delays in their initial pathway to initial cancer detection and treatment.

## 3 Measures

### 3.1 Pathway to Initial Cancer Detection and Treatment

In three open‐ended interview questions, participants were asked to recall whether they perceived any delays at three distinct steps: (i) seeking medical attention, (ii) receiving their diagnosis and (iii) commencing treatment in their pathway to initial cancer detection and treatment. If a participant reported perceiving a delay at any of these steps, they were prompted to provide further information on reasons for the perceived delay. Participants' responses were recorded during the interview on a paper interview guide by the interviewer and were subsequently entered into an online data file by a researcher post‐interview.

### 3.2 Demographic and Clinical Characteristics

Participants were asked to self‐report their age, sex, country of birth, highest education level, annual household income, access to private health insurance, marital status and cancer type. Residential street addresses were geocoded and mapped to the 2011 Statistical Area Level 2 (SA2) boundaries using MapMarker Australia Version 15.16.0.21 and MapInfo Pro Version 15.0 SA2. Geographical remoteness was determined using the Australian Bureau of Statistics Accessibility/Remoteness Index of Australia (ARIA), and relative area‐level disadvantage was identified using the Socio‐Economic Indexes for Areas (SEIFA) [23, 24]. Cancer type was self‐reported in the questionnaire and, where possible, verified against the population‐based Queensland Cancer Register (QCR). Self‐report data were relied upon where the diagnosis could not be verified by the QCR (*n* = 71, 10%).

### 3.3 Data Analysis

Study participants were included in this analysis if they had a confirmed cancer diagnosis and provided at least one response to one of the interview questions about perceived delays in the pathway to initial cancer detection and treatment. Descriptive statistics were used to summarise the sociodemographic characteristics of the study participants. Chi‐square tests and Fisher's exact T‐tests were used to compare the characteristics of participants included in this analysis with those excluded.

The number and proportion of participants who did and did not perceive a delay were calculated for each of the three steps: (i) seeking medical attention, (ii) receiving their diagnosis and (iii) commencing treatment. Additionally, the number and proportion of participants who perceived a delay at one, two or all steps were calculated. Descriptive statistics were used to summarise the sociodemographic characteristics of the participants who did and did not perceive a delay at each step. Chi‐square tests and Fisher's exact T‐tests were used to compare the characteristics of these groups of participants. If the difference between the two groups was statistically significant for variables with more than two categories (e.g., age and cancer type), post‐hoc chi‐square tests were conducted to compare each category between the two groups of participants. When determining statistically significant differences in post hoc analyses, a Bonferroni correction for multiple comparisons was applied.

Participants' descriptions of self‐reported reasons for perceived delays at each step were analysed using conventional content analysis, as described by Hsieh and Shannon, whereby codes and categories were derived inductively from the interview data rather than based on literature [25]. First, two authors (A.T.‐S. and I.B.) familiarised themselves with the data by reading through the interview responses several times. Second, for each of the three steps separately, responses were coded by one researcher (IB) using Microsoft Excel and documented in a coding framework using Microsoft Word, which included representative quotes alongside codes to illustrate the analytic findings. Then, codes were categorised based on the perceived source of delay: (i) personal (i.e., related to the person with cancer), (ii) healthcare professional or (iii) healthcare system (i.e., organisational processes, policies). Codes that did not fit into one of these categories (e.g., public holidays) were categorised as (iv) other. The coding framework was reviewed by a second researcher (A.T.‐S.) to ensure all codes were mutually exclusive before independently applying it to the full dataset. Any discrepancies in coding between the two researchers were resolved by a third researcher (E.J.). The final coding frameworks for each step are shown in Tables S2–, S4.

Concept maps were developed to visually summarise participants' perceived sources of delay and self‐reported reasons for these perceived delays at each of the three steps. For each of the three steps, descriptive statistics were used to summarise the number and proportion of participants reporting each category (i.e., personal, healthcare professional, healthcare system and other) as the perceived source of delay. The number and proportion of participants reporting each code (i.e., self‐reported reason for the perceived delay) were also summarised. Findings are presented below, alongside representative participant quotes to illustrate the analytic findings.

Finally, the number and proportion of participants whose perceived delay in commencing treatment (i.e., Step 3) was an actual delay were determined based on previous analyses conducted in this cohort [26]. Briefly, for participants with a relevant Optimal Cancer Pathway (OCP) for their cancer type and self‐reported dates for both diagnosis and treatment commencement, actual delays in commencing treatment were determined by comparing the number of weeks between diagnosis and starting treatment with recommended timeframes provided in the relevant OCP [27]. Participants whose timeframe between diagnosis and treatment commencement exceeded the number of weeks recommended by the relevant OCP (based on cancer type and modality of initial treatment) were classified as experiencing an actual delay in commencing treatment.

## 4 Results

### 4.1 Participant Characteristics

Of the 811 participants who consented to participate in the Travelling for Treatment study over the 4‐year study period, 686 (85%) were eligible for inclusion in this analysis (Figure S1). Participants who did not complete an interview at study recruitment (*n* = 99) or did not respond to any questions relating to delays in their pathway to initial cancer detection and treatment (*n* = 15), along with participants who were undergoing investigations for cancer but were not eventually diagnosed with cancer (*n* = 11) were excluded from this analysis. Compared to those excluded from this analysis, participants included in this analysis were more likely to be born overseas (*χ* ^2^(1) = 27.079, *p* = < 0.001, phi [*φ*] = 0.183) and have completed tertiary education (*χ* ^2^(1) = 5.827, *p* = 0.016, *φ* = 0.088). Participants included in this analysis were also more likely to be diagnosed with breast cancer (*χ* ^2^(1) = 10.176, *p* = < 0.001, *φ* = 0.113) and less likely to be diagnosed with cancers of unknown primary origin (*χ* ^2^(1) = 170.328, *p* = < 0.001, *φ* = 0.462) (Table S5).

Participant characteristics are shown in Table 1. Half of the participants (50%) in this analysis completed their interview within 6 months of receiving their cancer diagnosis. Participants' ages ranged from 26 to 93 years (median = 66 years, interquartile range = 58–72 years), 53% identified as male and 47% as female and 80% were born in Australia. The most common primary cancer diagnoses were breast (18%), head and neck (15%), prostate (12%) and skin (12%) cancer. Most participants lived in inner (45%) and outer (43%) regional areas. A small proportion of participants (4%) were classified as living in a major city; however, they were included in the study as they had travelled > 50 km to access cancer treatment or follow‐up care. Over two‐thirds of participants (68%) were in a relationship, de facto or married. Less than half (42%) had completed university or vocational level education. Participants' annual household income varied, with almost half (47%) earning under AU$30,000 per year. Most participants (79%) did not have private health insurance that partially or fully covered their cancer treatment.

**TABLE 1:** Sociodemographic characteristics of all participants and of participants who did and did not perceive a delay at three distinct steps in their pathway to initial cancer detection and treatment.

	Total sample (*N* = 686)		Seeking medical attention				Diagnosis				Commencing treatment			
			Perceived delay (*n* = 132)		No perceived delay (*n* = 554)		Perceived delay (*n* = 161)		No perceived delay (*n* = 525)		Perceived delay (*n* = 156)		No perceived delay (*n* = 530)	
	*n*	(%)^a^	*n*	(%)^a^	*n*	(%)^a^	*n*	(%)^a^	*n*	(%)^a^	*n*	(%)^a^	*n*	(%)^a^
Age (years)														
20–29	4	(1)	0	(0)	4	(1)	1	(1)	3	(1)	0	(0)	4	(1)
30–39	15	(2)	3	(2)	12	(2)	6	(4)	9	(2)	2	(1)	13	(3)
40–49	57	(8)	20	(15)	37	(7)	21	(13)	36	(7)	13	(8)	44	(8)
50–59	127	(19)	20	(15)	107	(20)	27	(32)	100	(19)	30	(19)	97	(19)
60–69	238	(35)	46	(35)	192	(35)	62	(39)	176	(34)	53	(35)	185	(35)
70+	232	(34)	41	(32)	191	(35)	41	(26)	191	(37)	55	(35)	177	(34)
Not reported	13		2		11		3		10		3		10	
Sex														
Male	355	(53)	62	(48)	293	(54)	77	(49)	278	(54)	71	(47)	284	(55)
Female	318	(47)	67	(52)	251	(46)	80	(51)	238	(46)	82	(53)	236	(45)
Not reported	13		3		10		4		9		3		10	
Country of birth														
Australia	548	(80)	107	(81)	441	(80)	132	(82)	416	(79)	123	(79)	425	(80)
Other	138	(20)	25	(19)	113	(20)	29	(18)	109	(21)	33	(21)	105	(20)
Highest level of education completed														
High school or lower	377	(58)	70	(56)	307	(58)	78	(51)	299	(60)	76	(51)	301	(60)
University/vocational	276	(42)	56	(44)	220	(42)	76	(49)	200	(40)	72	(49)	204	(40)
Not reported	33		6		27		7		26		8		25	
Annual household income^b^														
Under $30,000	297	(47)	54	(44)	243	(48)	65	(42)	232	(49)	66	(46)	231	(48)
$30,001–$50,000	109	(17)	24	(20)	85	(17)	29	(19)	80	(17)	29	(20)	80	(16)
$50,001–$80,000	79	(13)	14	(11)	65	(13)	21	(14)	58	(12)	12	(8)	67	(14)
$80,001–$100,000	33	(5)	4	(3)	29	(6)	9	(6)	24	(5)	11	(8)	22	(5)
Over $100,001	111	(18)	27	(22)	84	(17)	29	(19)	82	(16)	25	(17)	86	(18)
Not reported	57		9		48		8		49		13		44	
Private health insurance^c^														
Yes (full or partial coverage)	138	(21)	23	(19)	115	(22)	38	(25)	100	(20)	34	(23)	104	(21)
No	510	(79)	101	(81)	409	(78)	115	(75)	395	(80)	112	(77)	398	(79)
Not reported	38		8		30		8		30		10		28	
Marital status														
Single	97	(15)	17	(14)	80	(15)	23	(15)	45	(9)	22	(15)	75	(15)
In a relationship, de facto or married	444	(68)	86	(69)	358	(67)	102	(65)	342	(68)	103	(68)	341	(67)
Divorced	67	(10)	15	(12)	52	(10)	22	(14)	74	(15)	17	(11)	50	(10)
Widowed	49	(7)	7	(6)	42	(8)	9	(6)	40	(8)	9	(6)	40	(8)
Not reported	29		7		22		5		24		5		24	
Geographical remoteness (ARIA)														
Major city^d^	29	(4)	5	(4)	24	(4)	12	(7)	17	(3)	9	(6)	20	(4)
Inner regional	307	(45)	61	(46)	246	(45)	78	(48)	229	(44)	78	(50)	229	(44)
Outer regional	292	(43)	59	(45)	233	(42)	58	(36)	234	(45)	57	(37)	235	(45)
Remote or very remote	54	(8)	7	(5)	47	(9)	13	(8)	41	(8)	12	(8)	42	(8)
Not reported	4		—		4		—		4		—		4	
Area‐level disadvantage (SEIFA)														
Quintile 1 (lowest)	245	(36)	43	(33)	202	(37)	57	(35)	188	(36)	58	(37)	187	(36)
Quintile 2	206	(30)	42	(32)	164	(30)	44	(27)	162	(31)	49	(32)	157	(30)
Quintile 3	150	(22)	30	(23)	120	(22)	40	(25)	110	(21)	26	(17)	124	(24)
Quintile 4	74	(11)	15	(11)	59	(11)	18	(11)	56	(11)	23	(15)	51	(10)
Quintile 5 (highest)	7	(1)	2	(2)	5	(1)	2	(1)	5	(1)	0		7	(1)
Not reported	4		—		4		—		4		—		4	
Cancer type														
Breast	125	(18)	18	(14)	107	(19)	21	(13)	104	(20)	27	(17)	98	(19)
Colorectal	44	(6)	8	(6)	36	(6)	11	(7)	33	(6)	9	(6)	35	(7)
Gynaecological	61	(9)	22	(17)	39	(7)	22	(14)	39	(7)	20	(13)	41	(8)
Head and neck	105	(15)	22	(17)	83	(15)	23	(14)	82	(16)	16	(10)	89	(17)
Lung	49	(7)	10	(8)	39	(7)	10	(6)	39	(7)	11	(7)	38	(7)
Prostate	82	(12)	7	(5)	75	(14)	14	(9)	68	(13)	22	(14)	60	(11)
Skin	81	(12)	18	(14)	63	(11)	19	(12)	62	(12)	15	(10)	66	(12)
Other^e^	130	(19)	22	(17)	108	(19)	38	(24)	92	(18)	33	(21)	97	(18)
Unknown primary^f^	9	(1)	5	(4)	4	(1)	3	(2)	6	(1)	3	(2)	6	(1)

Abbreviations: ARIA, Accessibility/Remoteness Index of Australia; SEIFA, Socio‐Economic Indices for Areas.

^a^ Percentage calculated based on non‐missing data.

^b^ Australian Dollars (2017–2020, depending on date of study recruitment).

^c^ Private health insurance that covered (partially or fully) cancer treatment.

^d^ Participants classified as living in a major city according to ARIA (23) were included in this sample of rural cancer patients as they had travelled > 50 km for cancer care.

^e^ Includes anal, bladder, bone, brain, connective tissue/peripheral nerve, eye, gallbladder, kidney, lip, liver, oesophageal, other lymphatic, pancreatic, small intestine, stomach, testicular, thymus, heart, mediastinum and pleura and thyroid cancers, leukaemia, lymphoma, myelodysplastic disease, myeloma and non‐Hodgkin's lymphoma.

^f^ Participants with an unknown primary cancer site.

### 4.2 Perceived Delays in the Pathway to Initial Cancer Detection and Treatment

Almost half of the participants (*n* = 320, 47%) perceived a delay at one or more of the three steps: (i) seeking medical attention, (ii) receiving their diagnosis and (iii) commencing treatment. Of these, 206 (64%) participants perceived a delay at only one step, 99 (31%) perceived a delay at two steps and 15 (5%) perceived a delay at all three steps.

### 4.3 Perceived Delays in Seeking Medical Attention

Almost a fifth of participants (*n* = 132, 19%) perceived a delay in initially seeking medical attention related to their subsequent cancer diagnosis. Characteristics of participants who did and did not perceive a delay are shown in Table 1. Characteristics only significantly differed with respect to age (*χ* ^2^(5) = 11.499, *p* = 0.042, *φ*_*c*_ = 0.131) and cancer type (*χ* ^2^(8) = 27.867, *p* = < 0.001, *φ*_*c*_ = 0.202) (Table S6). Participants aged 40 to 49 years (*χ* ^2^(1) = 9.939, *p* = 0.002, *φ*_*c*_ = 0.122) were more likely to perceive a delay in seeking medical attention. Participants with gynaecological cancer (*χ* ^2^(1) = 12.195, *p* = < 0.001, *φ* = 0.133) and those with cancers of unknown primary origin (*χ* ^2^(1) = 7.739, *p* = < 0.005, *φ* = 0.106) were also more likely to perceive a delay in seeking medical attention (Table S6).

**FIGURE 1:**
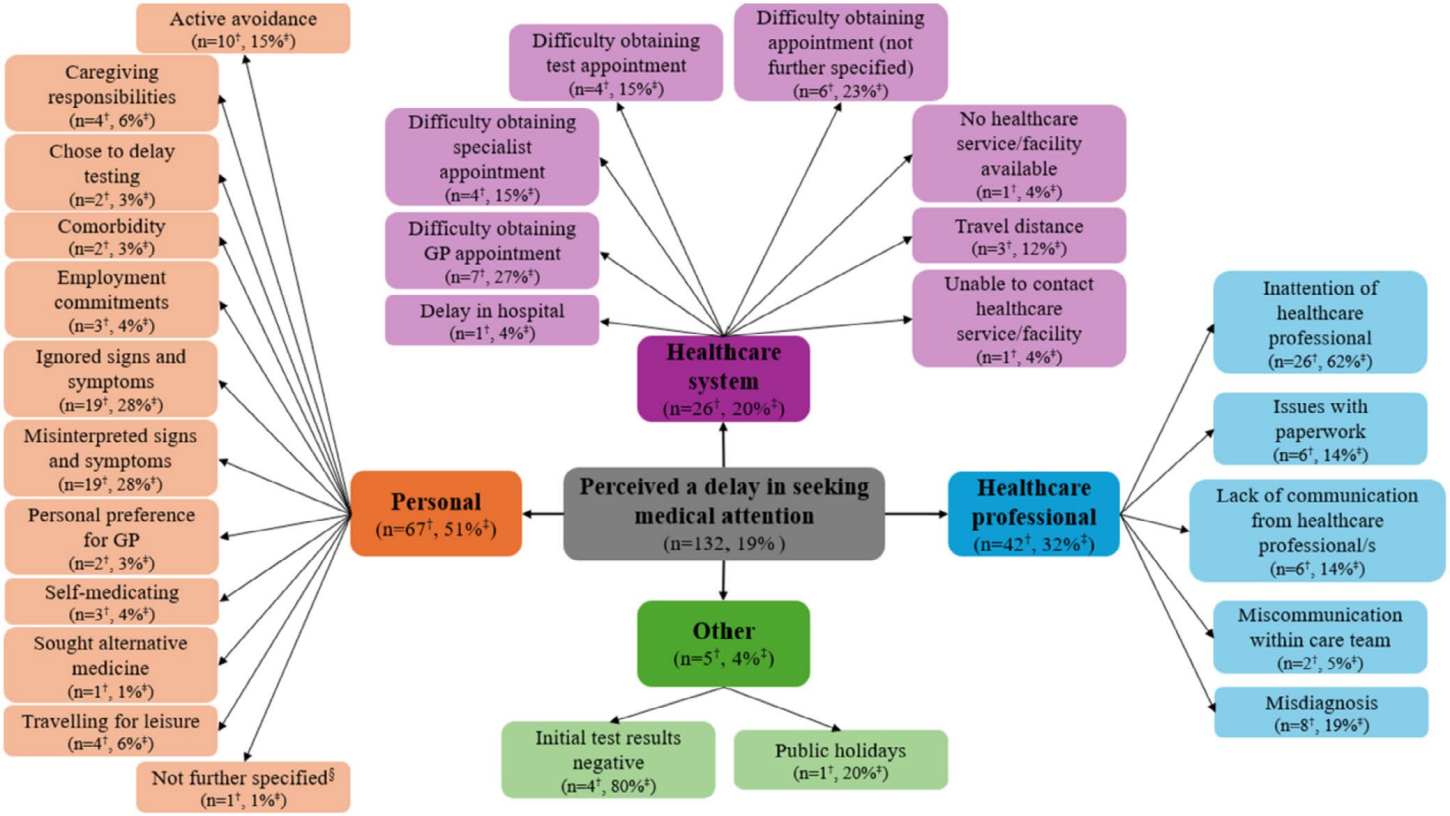
Concept map of participants' perceived sources of delay in seeking medical attention and self‐reported reasons for these perceived delays. ^†^ *n* for categories represents the number of participants who reported the category as a perceived source of delay. The sum of *n* exceeds the total number of participants (*n* = 132), as participants' responses may include content that was relevant to more than one category. *n* for codes represents the number of participants who reported the code (i.e., self‐reported reason for the perceived delay). The sum of *n* may exceed category *n* as participants' responses may include content that was relevant to more than one code within the category. ^‡^% for categories represents the proportion of participants who reported the category as a perceived source of delay. Sum of percentages exceeds 100% because participants' responses may include content that was relevant to more than one category. % for codes represents the proportion of participants who reported the code within each category. Sum of percentages may exceed 100% as participants' responses may include content that was relevant to more than one code within the category. ^§^Participant self‐reported reason for perceived delay was personal, but did not provide further information on why. GP, general practitioner.

Participants' self‐reported reasons for perceived delays in seeking medical attention are shown in Figure 1, with participant quotes presented in Table 2. Of the participants who perceived a delay in seeking medical attention, half (*n* = 67, 51%) perceived this was due to personal factors, such as misinterpreting (*n* = 19, 28%) or ignoring (*n* = 19, 28%) their signs and symptoms, travelling for leisure (*n* = 4, 6%) and competing caregiving responsibilities (*n* = 4, 6%) or employment commitments (*n* = 3, 4%). Almost one‐third (*n* = 42, 32%) perceived that their delay was related to factors associated with healthcare professionals. For example, participants perceived inattention from the healthcare professional (*n* = 26, 62%) with multiple consultations required before their concern (i.e., their cancer‐related symptom/s) was addressed (i.e., via testing or referrals to specialists). In other cases, participants reported a misdiagnosis (*n* = 8, 19%) by the healthcare professional, which resulted in action and/or treatment undertaken for the misdiagnosed condition. Participants perceived these to delay their efforts in seeking medical attention for their current cancer, including delays in obtaining referrals for cancer‐related testing or specialist consultation. Some participants (*n* = 26, 20%) perceived that their delay was related to factors associated with the healthcare system, particularly difficulties in obtaining appointments with general practitioners (*n* = 7, 27%) or specialists (*n* = 4, 15%) and at cancer screening or testing facilities, such as mammograms (*n* = 4, 15%). A small number of participants perceived that their delay was due to other reasons (*n* = 5, 4%), such as initially receiving negative test results (*n* = 4, 80%) or the impact of public holidays such as the Christmas and New Year's period (*n* = 1, 20%).

**TABLE 2:** Self‐reported reasons for perceived delays by participants who perceived a delay in seeking medical attention (*n* = 132).

Self‐reported reason for perceived delay	Example of participant quotes for each category
Personal	‘I was supposed to have mammogram done 1 year ago but daughter‐in‐law got breast cancer which metastasised, and I was looking after her’. (P10465, F, 60–69 years, inner regional, breast) ‘Work is hard to cover…’ (P14089, M, 60–69 years, outer regional, other^a^) ‘I thought it would go away’. (P10081, M, 50–59 years, outer regional, head and neck) ‘I thought it was just a lump, then a friend told me to get it checked’. (P10065, F, 50–59 years, outer regional, skin) ‘My usual GP left after 10 years, and I found it hard to find another GP that I trusted…’ (P10501, F, 40–49 years, outer regional, breast) ‘Treating with over‐the‐counter medication’. (P10231, M, 60–69 years, inner regional, other^a^) ‘Travelling abroad’. (P10038, M, 70+ years, inner regional, skin)
Active avoidance	
Caregiving responsibilities	
Chose to delay testing	
Comorbidity	
Employment commitments	
Ignored signs and symptoms	
Misinterpreted signs and symptoms	
Personal preference for GP	
Self‐medicating	
Sought alternative medicine	
Travelling for leisure	
Not further specified^c^	
Healthcare professional	‘Had been to doctors twice before with same symptoms’. (P10328, M, 70+ years, outer regional, other^a^) ‘Was supposed to have six‐month check‐ups but fell through the cracks and no‐one followed up’. (P12141, M, 70+ years, outer regional, lung) ‘[My cancer] was misdiagnosed as heart burn by GP’. (P10382, M, 70+ years, outer regional, other) ‘Saw the GP as I was concerned about ulcer under tongue. GP dismissed it and it was not until a dental exam that the dentist knew something was wrong’. (P12026, M, 60–69 years, inner regional, skin) ‘It took visits once a month for 3 months before I was referred for scans’. (P12546, F, 70+ years, outer regional, head and neck)
Inattention of healthcare professional	
Issues with paperwork	
Lack of communication from healthcare professional/s	
Miscommunication within care team	
Misdiagnosis	
Healthcare system	‘[Regional city] only had two doctors, could not be fit in for 2 weeks when I had symptoms’. (P12248, F, 70+ years, inner regional, gynaecological) ‘Waited 3 months to see a specialist’. (P12541, M, 70+ years, inner regional, skin) ‘It was difficult to get in for a mammogram, put on waiting list’. (P10141, F, 40–49 years, outer regional, breast) ‘Going through public system and it took so long’. (P15180, F, 60–69 years, outer regional, gynaecological) ‘Long travel time to GP’. (P14032, F, 70+ years, remote or very remote, other^a^) ‘Calls from BreastScreen were ‘no caller ID’ so couldn't ring them back’. (P14198, F, 70+ years, inner regional, breast)
Delay in hospital	
Difficulty obtaining GP appointment	
Difficulty obtaining specialist appointment	
Difficulty obtaining test appointment	
Difficulty obtaining appointment (not further specified)	
No healthcare service/facility available	
Travel distance	
Unable to contact healthcare service/facility	
Other	‘First test was negative, so thought things were okay. Symptoms did not go away so went back to GP’. (P12124, M, 70+ years, major city^b^, head and neck) ‘Sent to a radiology clinic for a scan and they gave me the ‘all clear’ and could not find anything wrong’. (P12504, M, 70+ years, inner regional, head and neck) ‘Delayed going to GP due to Christmas and New Year period’ (P12310, M, 60–69 years, inner regional, head and neck)
Initial test results negative	
Public holidays	

*Note:* Participant characteristics denoted in brackets after quotes by participant identification number, sex (M = male or F = female), age bracket (years), geographical remoteness and cancer type.

Abbreviation: GP, general practitioner.

^a^ Includes anal, bladder, bone, brain, connective tissue/peripheral nerve, eye, gallbladder, kidney, lip, liver, oesophageal, other lymphatic, pancreatic, small intestine, stomach, testicular, thymus, heart, mediastinum and pleura and thyroid cancers, leukaemia, lymphoma, myelodysplastic disease, myeloma and non‐Hodgkin's lymphoma.

^b^ Participants classified as living in a major city according to Accessibility/Remoteness Index of Australia (23) were included in this sample of rural cancer patients as they had travelled > 50 km for cancer care.

^c^ Participant self‐reported reason for perceived delay was personal, but did not provide further information on why.

### 4.4 Perceived Delays in Receiving Their Cancer Diagnosis

Almost a quarter of participants (*n* = 161, 23%) perceived a delay in receiving their diagnosis. Characteristics of participants who did and did not perceive a delay in diagnosis are shown in Table 1. Characteristics only significantly differed with respect to age (*χ* ^2^(5) = 13.528, *p* = 0.019, *φ*_*c*_ = 0.142), geographical remoteness (*χ* ^2^(3) = 7.907, *p* = 0.048, *φ*_*c*_ = 0.108) and highest level of education completed (*χ* ^2^(1) = 4.145, *p* = 0.042, *φ* = 0.080) (Table S6). Participants who perceived a delay in receiving their diagnosis were more likely to have completed university or vocational education compared to those who did not perceive a delay. While the overall chi‐square tests identified a significant association between delay status and both age and geographical remoteness, no specific age category or geographical area showed a statistically significant difference after controlling for multiple comparisons (Table S6). Thus, the difference observed between those who did and those who did not perceive a delay may not be attributable to any one category within each variable, but rather to the overall distribution of ages and geographical remoteness.

**FIGURE 2:**
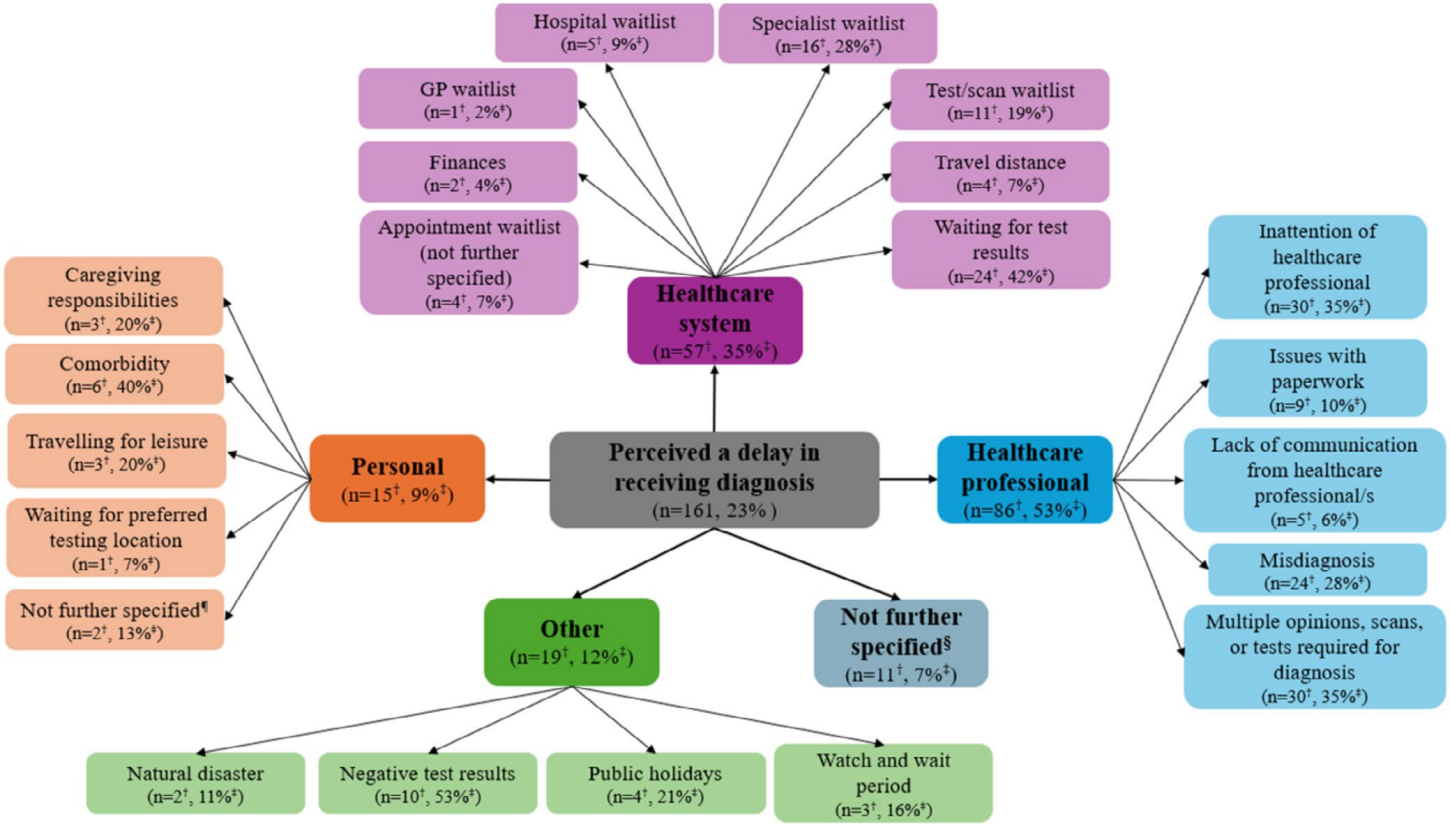
Concept map of participants' perceived sources of delay in receiving their cancer diagnosis and self‐reported reasons for this perceived delay. ^†^ *n* for categories represents the number of participants who reported the category as a perceived source of delay. The sum of *n* exceeds the total number of participants (*n* = 161) as participants' responses may include content that was relevant to more than one category. *n* for codes represents the number of participants who reported the code (i.e., self‐reported reason for the perceived delay). The sum of *n* may exceed category *n* as participants' responses may include content that was relevant to more than one code within the category. ^‡^% for categories represents the proportion of participants who reported the category as a perceived source of delay. Sum of percentages exceeds 100% because participants' responses may include content that was relevant to more than one category. % for codes represents the proportion of participants who reported the code within each category. Sum of percentages may exceed 100% as participants' responses may include content that was relevant to more than one code within the category. ^§^Participant perceived a delay in receiving their diagnosis but did not self‐report on reasons why. ^¶^Participant self‐reported reason for perceived delay was personal, but did not provide further information on why. GP, general practitioner.

Participants' self‐reported reasons for perceived delays in receiving their cancer diagnosis are shown in Figure 2, with participant quotes presented in Table 3. Over half (*n* = 86, 53%) perceived that the delay was related to their interactions with, or the actions of, healthcare professionals. For example, the healthcare professional required multiple opinions or further tests or scans prior to confirming the diagnosis (*n* = 30, 35%) or being initially misdiagnosed (*n* = 24, 28%). Around one third of participants (*n* = 57, 35%) perceived delays due to challenges accessing the healthcare system, including waiting lists for specialists (*n* = 16, 28%) and tests or scans (*n* = 11, 19%). In some cases (*n* = 15, 9%), participants perceived that the delay in receiving their diagnosis was due to personal reasons, such as their comorbidities (*n* = 6, 40%), competing caregiving responsibilities (*n* = 3, 20%) and travelling for leisure (*n* = 3, 20%). Other delays (*n* = 19, 12%) included the perceived impact of natural disasters (*n* = 2, 11%), public holidays (*n* = 4, 21%) and receiving negative test results (*n* = 10, 53%). A small number of participants (*n* = 11, 7%) perceived that they were delayed in receiving their diagnosis, but did not provide further information on reasons why.

**TABLE 3:** Self‐reported reasons for perceived delays by participants who perceived delay in receiving their cancer diagnosis (*n* = 161).

Self‐reported reason for perceived delay	Example participant quotes for each category
Healthcare professional	‘Went back and forward for no reason. I should have been referred’. (P10088, M, 70+ years, inner regional, skin) ‘Different specialists were unsure if it was cancer or not’. (P10049, M, 50–59 years, inner regional, other^a^) ‘Paperwork was lost between doctor and hospital’. (P10335, M, 30–39 years, outer regional, other^a^) ‘Radiologist misdiagnosed it as a cyst twice. GP sent for a biopsy and then it was diagnosed as cancer’. (P12126, F, 30–39 years, inner regional, breast) ‘GP did not pass on referral to specialist’. (P14065, M, 70+ years, outer regional, prostate)
Inattention of healthcare professional	
Issues with paperwork	
Lack of communication from healthcare professional/s	
Misdiagnosis	
Multiple opinions, scans or tests required for diagnosis	
Healthcare system	‘Surgeon in [Regional City] could not operate (to investigate) as private hospital was not aligned with [my] private health fund so had to go to [Major City]’. (P10130, M, 60–69 years, inner regional, unknown primary^b^) ‘ENT doctor was going to do a biopsy but could not do it because there were no beds available in ICU’. (P12104, M, 50–59 years, inner regional, head and neck) ‘Remoteness from specialist practice’. (P10018, M, 30–39 years, remote or very remote, skin) ‘Had to travel for mammogram’. (P15103, F, 60–69 years, outer regional, breast) ‘Took 3 months to get an appointment at the hospital. Scheduled appointments were cancelled twice’. (P12425, F, 60–69 years, inner regional, gynaecological)
Appointment waitlist (not further specified)	
Finances	
GP waitlist	
Hospital waitlist	
Specialist waitlist	
Test/scan waitlist	
Travel distance	
Waiting for test results	
Other	‘Had tests which came back negative’. (P12153, F, 70+ years, inner regional, gynaecological) ‘Christmas and New Years got in the way to do the procedure’. (P10363, F, 40–49 years, major city^c^, gynaecological) ‘[Hospital department staff] wanted to wait and see’. (P1259, M, 20–29 years, inner regional, head and neck)
Natural disaster	
Negative test results	
Public holidays	
Watch and wait period	
Personal	‘Waited 1 week to receive results because I went to [City]’. (P15102, F, 40–49 years, remote or very remote, breast) ‘Endoscopy which diagnosed cancer was delayed a few days due to comorbidity’. (P10116, M, 60–69 years, inner regional, other^a^) ‘I thought I was too busy to be sick… I had to take care of my granddaughter’. (P10310, F, 70+ years, remote or very remote, gynaecological)
Caregiving responsibilities	
Comorbidity	
Travelling for leisure	
Waiting for preferred testing location	
Not further specified^d^	
Not further specified^e^	

*Note:* Participant characteristics denoted in brackets after quotes by participant identification number, sex (M = male or F = female), age bracket (years), geographical remoteness and cancer type.

Abbreviations: ENT: ear, nose and throat. ICU: intensive care unit, GP: General Practitioner.

^a^ Includes anal, bladder, bone, brain, connective tissue/peripheral nerve, eye, gallbladder, kidney, lip, liver, oesophageal, other lymphatic, pancreatic, small intestine, stomach, testicular, thymus, heart, mediastinum and pleura and thyroid cancers, leukaemia, lymphoma, myelodysplastic disease, myeloma and non‐Hodgkin's lymphoma.

^b^ Diagnosed with cancer of an unknown primary cancer site.

^c^ Participants classified as living in a major city according to the Accessibility/Remoteness Index of Australia (23) were included in this sample of rural cancer patients as they had travelled > 50 km for cancer care.

^d^ Participant self‐reported reason for perceived delay was personal, but did not provide further information on why.

^e^ Participant perceived a delay in receiving their diagnosis but did not self‐report on reasons why.

### 4.5 Perceived Delays in Commencing Treatment

Delays in commencing treatment were perceived by almost a quarter (*n* = 157, 23%) of participants. Characteristics of participants who did and did not perceive a delay in commencing treatment are shown in Table 1, with no statistically significant differences observed between the two groups (Table S6).

**FIGURE 3:**
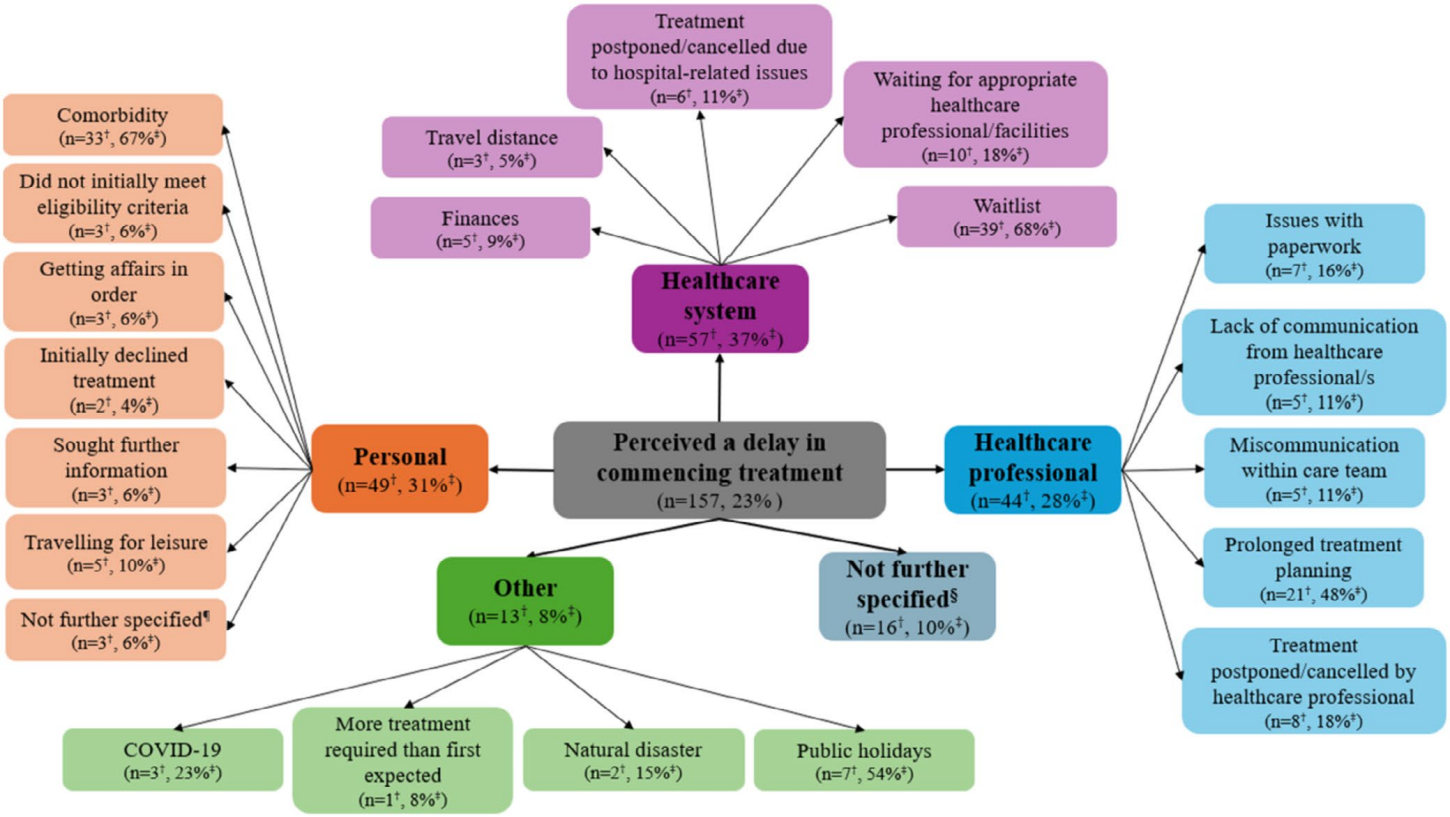
Concept map of participants' perceived sources of delay in commencing treatment and self‐reported reasons for these perceived delays. ^†^ *n* for categories represents the number of participants who reported the category as a perceived source of delay. Sum of *n* exceeds the total number of participants (*n* = 157) as participants' responses may include content that was relevant to more than one category. *n* for codes represents the number of participants who reported the code (i.e., self‐reported reason for the perceived delay). Sum of n may exceed category *n* as participants' responses may include content that was relevant to more than one code within the category. ^‡^% for categories represents the proportion of participants who reported the category as a perceived source of delay. Sum of percentages exceeds 100% because participants' responses may include content that was relevant to more than one category. % for codes represents the proportion of participants who reported the code within each category. Sum of percentages may exceed 100% as participants' responses may include content that was relevant to more than one code within the category. ^§^Participant perceived a delay in receiving their cancer diagnosis but did not self‐report on the reasons why. ^¶^Participant self‐reported reason for perceived delay was personal, but did not provide further information on why.

Participants' self‐reported reasons for perceived delays in commencing cancer treatment are shown in Figure 3, with participant quotes presented in Table 4. More than one third (*n* = 57, 37%) perceived the delay to be related to challenges accessing the healthcare system, particularly waitlists for treatment (*n* = 39, 68%), waiting for the appropriate healthcare professional or healthcare facility (*n* = 10, 18%) and travel distances to treatment facilities (*n* = 3, 5%). Almost one third (*n* = 49, 31%) perceived the delay was related to personal factors, such as their comorbidities delaying commencement (*n* = 33, 67%) or their personal choices regarding treatment, including seeking further information (*n* = 3, 6%) or initially declining treatment (*n* = 2, 4%). Over a quarter of participants (*n* = 44, 28%) perceived the delay in commencing treatment was related to their interactions with, or actions of, healthcare professionals, for example, prolonged treatment planning (*n* = 21, 48%), having treatment rescheduled or cancelled due to the healthcare professionals' personal commitments (*n* = 8, 18%), or miscommunications within the care team (*n* = 5, 11%). Other delays (*n* = 13, 8%) included the perceived impact of natural disasters (*n* = 2, 15%) and public holidays (*n* = 7, 54%). A small number of participants (*n* = 16, 10%) perceived that they were delayed in commencing treatment, but did not provide further information on reasons why.

**TABLE 4:** Self‐reported reasons for perceived delays by participants who perceived a delay in commencing treatment (*n* = 157).

Self‐reported reason for perceived delay	Example participant quotes for each category
Healthcare system	‘Could not afford to pay private so was unable to accept next surgery date. Had to go on public waitlist’. (P10149, M, 60–69 years, inner regional, prostate) ‘Nurse/hospital beds not available… surgery was delayed 2 weeks’. (P10185, M, 50–59 years, inner regional, other^a^) ‘[Regional hospital] did not have appropriate equipment’. (P10159, F, 70+ years, outer regional, breast) ‘Appointments made and cancelled by hospital’. (P14008, F, 60–69 years, outer regional, other^a^) ‘Told that there were many oncologists away. [Therefore, they were] seeing more urgent cases first’. (P14072, F, 50–59 years, remote or very remote, breast)
Finances	
Travel distance	
Treatment postponed/cancelled due to hospital‐related issues	
Waiting for appropriate healthcare professional/facilities	
Waitlist	
Personal	‘Blood pressure issue that needed to be dealt with first’. (P10001, M, 70+ years, inner regional, other^a^) ‘Had to go back home just to get affairs in order’. (P10052, M, 70+ years, outer regional, other^a^) ‘Didn't want chemo[therapy]’. (P10072, F, 50–59 years, inner regional, other^a^) ‘Could have gone sooner but had a planned trip overseas’. (P10099, M, 70+ years, inner regional, prostate) ‘Spent months doing research and conducting alternative therapies’. (P14000, F, 60–69 years, inner regional, other^a^)
Comorbidity	
Did not initially meet eligibility criteria	
Getting affairs in order	
Initially declined treatment	
Sought further information	
Travelling for leisure	
Not further specified^b^	
Healthcare professional	‘Doctors could not decide what to do’. (P12076, F, 50–59 years, inner regional, colorectal) ‘I was told I needed surgery within 30 days. Next, I was told surgeon was going on holidays for 6 weeks—date was pushed out again’. (P10491, M, 60–69 years, inner regional, prostate) ‘[Hospital 1] delayed [sending] paperwork to [Hospital 2] [which] caused treatment start date 8 weeks after diagnosis’. (P14059, F, 30–39 years, inner regional, colorectal)
Issues with paperwork	
Lack of communication from healthcare professional/s	
Miscommunication within care team	
Prolonged treatment planning	
Treatment postponed/cancelled by healthcare professional	
Other	‘COVID‐19 interfered… Specialist doctors were not travelling’. (P12586, F, 60–69 years, outer regional, gynaecological) ‘Cyclone’. (P10121, F, 50–59 years, inner regional, breast) ‘Because of all the public holidays, [we] could not find surgery times’. (P12189, F, 50–59 years, inner regional, head and neck)
COVID‐19	
More treatment required than first expected	
Natural disaster	
Public holidays	
Not further specified^c^	

*Note:* Participant characteristics denoted in brackets after quotes by participant identification number, sex (M = male or F = female), age bracket (years), geographical remoteness and cancer type.

Abbreviation: GP, General Practitioner.

^a^ Includes anal, bladder, bone, brain, connective tissue/peripheral nerve, eye, gallbladder, kidney, lip, liver, oesophageal, other lymphatic, pancreatic, small intestine, stomach, testicular, thymus, heart, mediastinum and pleura and thyroid cancers, leukaemia, lymphoma, myelodysplastic disease, myeloma and non‐Hodgkin's lymphoma.

^b^ Participant self‐reported reason for perceived delay was personal, but did not provide further information on why.

^c^ Participant perceived a delay in commencing treatment but did not self‐report on reasons why.

### 4.6 Perceived Versus Actual Delays in Commencing Treatment

Of the 157 participants in this analysis who perceived a delay in commencing treatment, 108 (69%) had data available on actual treatment delay status, with over half (*n* = 62, 57%) experiencing a treatment delay according to the relevant Optimal Care Pathways for their cancer type based on their treatment modality.

## 5 Discussion

Almost half of rural cancer survivors perceived a delay at some point in the pathway to initial cancer detection and treatment. Self‐reported reasons for perceived delays were broadly related to personal factors, the interactions with or actions of, healthcare professionals and the complexities of the healthcare system. However, the diverse experiences within each of these broad categories indicate that a multitude of factors impact rural cancer survivors. Most commonly, self‐reported reasons for perceived delays in seeking medical attention were related to personal factors, perceived delays in receiving their diagnosis were related to interactions with or actions of, healthcare professionals and perceived delays in commencing treatment were related to healthcare system factors.

This attribution pattern aligns with attribution theory, whereby individuals seek logical explanations for events based on their perceived causality and control [28]. These attributions likely reflect participants' lived experiences, as individuals are most aware of their own behaviours during help‐seeking, rely on healthcare professionals for diagnoses and depend on healthcare system's efficiency for timely initiation of treatment. However, it is important to recognise that these attributions may oversimplify the interplay of factors contributing to delays. For example, personal delays may be compounded by a lack of accessible health education, diagnostic delays due to systemic constraints on healthcare providers in rural areas and treatment delays by broader structural inequities within the urban–rural divide. This highlights the need for interventions that address both the individual, organisational and systemic barriers to timely care in the pathway to initial cancer detection and treatment.

Personal factors were common causes of perceived delays among rural cancer survivors in this cohort, particularly in the initial stages of seeking medical attention. It is well known that the time taken for survivors to assess their symptoms and decide to seek care is a key factor in timely detection, diagnosis and treatment of cancer [1, 2, 29]. Help‐seeking behaviour represents a complex decision‐making process since individual decisions do not occur in isolation and are often shaped by systemic factors, such as limited access to healthcare facilities, low health literacy and sociocultural norms surrounding healthcare utilisation [30]. While participants may have been more likely to perceive delays due to personal factors, the interplay between these factors underscores the importance of ensuring public health initiatives address the broader inequities and barriers that influence the behaviours of rural cancer survivors.

Rural cancer survivors in this analysis commonly reported misinterpreting or ignoring signs and symptoms as having contributed to perceived delays in seeking medical attention. This has also been reported in qualitative studies of melanoma and lung cancer survivors in the United Kingdom (UK), with symptoms often misinterpreted and attributed to environmental (e.g., air conditioning) or physical (e.g., ageing, minor injuries) factors [31, 32]. This highlights the importance of increasing health literacy to improve knowledge regarding symptom identification, particularly recognition of symptoms that require medical attention. Personal factors, such as choosing to delay testing, caregiving responsibilities and employment commitments, may also contribute to the generally low participation in cancer screening programs that are seen across rural areas [33, 34]. Thus, addressing these barriers may support increased rural participation in cancer screening programs. Evaluation of programs aimed at increasing participation in Australia's National Bowel Cancer Screening Program has highlighted the importance of targeted awareness and education programs to specific subgroups, such as rural and remote communities [35].

Rural cancer survivors' interaction with, or the actions of, healthcare professionals was often reported as contributing to perceived delays in receiving their cancer diagnosis. A previous mixed methods study of rural cancer survivors in Australia identified that those with non‐specific symptoms (e.g., fatigue, weight loss, shoulder pain) often required the development of alarming symptoms (e.g., breast lump, haemoptysis), deterioration of current symptoms or identification of a family history of cancer to prompt further investigation and/or referrals [17]. Focus group discussions with general practitioners based in two rural Australian locations suggested that the compounding effect of ageing and presenting with several medical problems exacerbates the complexity of appointments and increases the likelihood of misdiagnosis [36]. Together with our findings, this highlights the difficulties that healthcare professionals face in balancing under‐ and over‐investigation of patient symptoms. It has been suggested that the use of cancer risk assessment tools, such as QCancer [37] used in the UK, may be beneficial in rural primary practice to assess a patient's cancer risk estimate by considering current symptoms and known cancer risk factors [38].

Healthcare system factors, particularly long waitlists, were commonly perceived as delays to commencing cancer treatment among this cohort of rural cancer survivors. In recent years, there has been an increase in funding and advocacy for treatment infrastructure in regional areas; for example, the introduction of tele‐oncology has allowed people to receive cancer care (i.e., systemic therapies) closer to home through local healthcare clinics under the remote supervision of specialists [39, 40]. People undergoing treatment for cancer report positive experiences with tele‐oncology, including receipt of high‐quality care, minimal disruptions to local support systems such as family and friends and reduction in financial, physical and emotional burden associated with traveling for treatment [39, 40]. However, several unintended consequences of introducing tele‐oncology need to be considered. For example, the increased workload on rural healthcare professionals may contribute to burnout, impacting recruitment and retention of healthcare professionals in rural areas, which could further exacerbate delays in care [41]. A qualitative study of rural cancer nurses in Australia highlighted the difficulties already experienced by rural healthcare professionals, such as significant workforce shortages, lack of skilled healthcare professionals and lack of telehealth preparedness [42]. Thus, the benefits of implementing technology, such as tele‐oncology, for improving access to cancer care for rural cancer survivors must also consider the potentially negative impacts upon the rural healthcare workforce and care pathways.

An important consideration for the current analysis is that not all perceived delays related to factors associated with healthcare professionals or the healthcare system are avoidable, and they may not reflect poor clinical performance. For example, healthcare professionals may require multiple tests or scans to confirm a diagnosis and time is required to process the results of tests or scans and navigate complex clinical cases with team discussions [43]. It is possible that healthcare professionals are acting, and treatment is commencing within recommended timeframes (i.e., within the recommended timeframes outlined in the Optimal Care Pathways [OCPs]), yet rural cancer survivors still perceived a delay. Just under half of the participants in this analysis who perceived a delay in commencing treatment did not face a treatment delay according to the OCPs. This raises the question about the level of discussion between patients and clinicians about treatment timelines, survivors' knowledge of where to access support to understand the treatment process and their access to resources, such as the OCPs. Overall, our findings highlight the need for, and importance of, clear patient‐clinician communication regarding the processes involved in diagnosis and treatment planning, particularly where survivors may be required to undergo further testing, wait for results or wait for treatment commencement.

This analysis looked at participants' self‐reported reasons for perceived delays at each of the three steps in their pathway to initial cancer detection and treatment. However, it is possible that a delay at an earlier step may have contributed to a delay at a later step, potentially impacting cancer stage at diagnosis, treatment options and response and survival [44]. As observed in our analysis, 36% of rural cancer survivors in this cohort perceived a delay to have occurred in more than one step in their pathway to initial cancer detection and treatment. Previous research has demonstrated the impact of compounding delays in cancer care, with a four‐week delay in commencing cancer treatment associated with increased mortality for several cancers [4]. Thus, the steps included in this analysis represent opportunities for early intervention to minimise the impact of compounding delays and improve overall survival outcomes.

### 5.1 Implications for Policy and Practice

In Australia, Optimal Care Pathways (OCPs) are available for 24 cancer types, with the implementation of the OCPs a key objective of the newly launched Australian Cancer Plan [45]. Population data show a positive correlation between the use of OCP‐aligned care and improved cancer outcomes [46]. In line with findings from our analysis, implementation of the OCPs may contribute to improved care for cancer survivors living in rural and remote areas by reducing delays in cancer care, especially for diagnosis and treatment [46]. Further, recent funding awarded for the development of an OCP specifically tailored for cancer survivors in rural and remote areas is a key step in reducing diagnosis and treatment delays among this cohort [47]. Findings from the current analysis provide insights into factors that may underlie or contribute to suboptimal care for rural cancer patients that could be addressed in the rural‐specific OCP. Additionally, the findings also highlight the importance of improved clinician‐patient communication regarding recommended timeframes, particularly for diagnosis and treatment, and survivors' knowledge of and access to the OCPs. With an estimated 169,500 cases of cancer diagnosed in Australia in 2024, and almost one‐third of Australians living in rural and remote areas, improving rural cancer care has the potential to benefit more than 55,000 people each year [5, 48].

### 5.2 Strengths and Limitations

This analysis included a large sample of rural cancer patients with a diverse range of cancer diagnoses. To our knowledge, this is one of the first studies to investigate rural cancer survivors' self‐reported reasons for perceived delays in their pathway to initial cancer detection and treatment. A key limitation of this study is that self‐reported reasons for perceived delays were not systematically assessed, rather were reported by participants in response to an open‐ended question (i.e., ‘If yes, why?’). Therefore, not every reason or their true prevalence may have been captured. Furthermore, due to the wording of the question and how participants' responses were recorded, perceived delays relating to seeking medical attention could not be categorised as delays in the patient interval (i.e., from first symptom to first visit to a healthcare professional) or the primary care interval (i.e., from first visit to a healthcare professional to referral).

Additionally, all participants in this study had accessed subsidised accommodation in a major city during their treatment or follow‐up care; therefore, compared to the broader population of rural cancer survivors, participants in this study may have been less likely to perceive delays in receiving their diagnosis and/or commencing treatment. The sample also only included a very small number of cancer survivors from remote or very remote areas (4%), and it is likely that those living in these areas would face additional challenges in seeking medical attention and accessing healthcare that may not have been fully captured in this analysis. Finally, it is not known whether the self‐reported reasons for perceived delays in this study are rural‐specific or applicable to cancer survivors more generally.

## 6 Conclusion

In a large cohort of rural cancer survivors in Australia, almost half perceived a delay in at least one step of their cancer care, from seeking medical attention to receiving their diagnosis and starting initial treatment. Perceived delays were broadly related to personal factors, the interactions with or actions of healthcare professionals and the complexities of the healthcare system. These findings identify several opportunities for improving the delivery of optimal care for rural cancer survivors. Main causes of perceived delays varied across the three steps of initial cancer detection and treatment, with personal factors the main cause of perceived delays for seeking medical attention; interactions with or actions of healthcare professionals for perceived delays in diagnosis; and healthcare system factors for perceived delays in commencing cancer treatment. Findings from this analysis highlight the need for improved patient–clinician communication, ongoing work to improve access to diagnostic and treatment infrastructure, and the implementation of Optimal Care Pathways in rural healthcare services. Continued efforts are also needed for the promotion of early help‐seeking and participation in cancer screening in rural areas.

## Author Contributions

**Alyssa Taglieri‐Sclocchi:** formal analysis, writing – review and editing, writing – original draft. **Ingrid Bindicsova:** formal analysis, writing – review and editing. **Susannah K. Ayre:** writing – original draft, writing – review and editing. **Michael Ireland:** conceptualization, methodology, writing – review and editing. **Sonja March:** conceptualization, methodology, writing – review and editing. **Fiona Crawford‐Williams:** conceptualization, methodology, data curation, writing – review and editing. **Suzanne Chambers:** conceptualization, methodology, writing – review and editing. **Jeff Dunn:** conceptualization, methodology, writing – review and editing. **Belinda C. Goodwin:** conceptualization, methodology, writing – review and editing. **Elizabeth A. Johnston:** methodology, supervision, writing – original draft, writing – review and editing.

## Ethics Statement

Ethical approval for this study was granted by the Human Research Ethics Committee of the University of Southern Queensland (H17REA152).

## Conflicts of Interest

The authors declare no conflicts of interest.

## Supporting information


Figure S1.

Table S1.

Table S2.

Table S3.

Table S4.

Table S5.

Table S6.

## Data Availability

The data that support the findings of this study are available from the corresponding author upon reasonable request.
