# Supplementary material for: Rural Cancer Survivors' Perceived Delays in Seeking Medical Attention, Diagnosis and Treatment: Findings From a Large Qualitative Study
Source: Cancer Med. 2025 Jul 21;14(14):e71036. doi: 10.1002/cam4.71036 (PMC12278023; doi:10.1002/cam4.71036)
Supplement: Supplementary file 1 — Figure S1. [file CAM4-14-e71036-s004.docx]

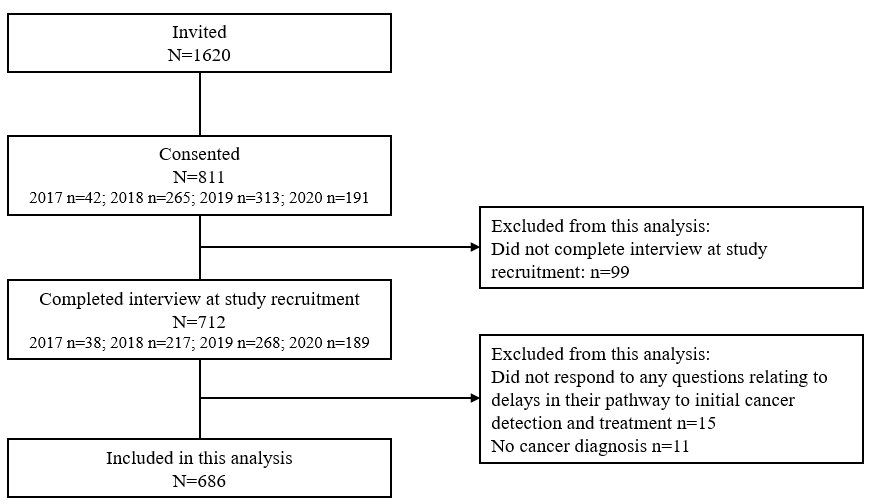


**Supplementary Fig. 1** Flowchart showing recruitment of rural cancer survivors to the Travelling for Treatment study, a longitudinal cohort study of rural cancer survivors and their caregivers, and those eligible for inclusion in this analysis of participants’ self-reported reasons for perceived delays in the pathway to initial cancer detection and treatment.
