# Supplementary material for: Rural Cancer Survivors' Perceived Delays in Seeking Medical Attention, Diagnosis and Treatment: Findings From a Large Qualitative Study
Source: Cancer Med. 2025 Jul 21;14(14):e71036. doi: 10.1002/cam4.71036 (PMC12278023; doi:10.1002/cam4.71036)
Supplement: Supplementary file 3 — Table S2. [file CAM4-14-e71036-s006.docx]

**Supplementary Table 2.** Coding framework for responses to the question “Did you feel like your seeking of medical attention was delayed in any way? If yes, why?”

| **Category** | **Code** | **Definition / Rule** | **Example/s** |
| --- | --- | --- | --- |
| Personal | Active avoidance | Participant aware of the issue but actively avoiding seeking medical attention. | 10135: “Put it off because didn’t want to know.” |
|  | Caregiving responsibilities | Participant reports taking care of family and/or friends. | 10465: “I was supposed to have mammogram done one year ago but daughter in law got breast cancer which metastasised, and I was looking after her.” |
|  | Chose to delay testing | Participant reports that they made a personal choice to delay testing but did not provide a specific reason that could be coded under another code. | 12256: “Moving around and didn’t get tests done for five to six years.” |
|  | Comorbidity | Participant reports a comorbidity was responsible for the delay such as an illness. | 10095: “Other cancer diagnosis had previously been given, impacted delay.” |
|  | Ignored signs and symptoms | Participant reports being aware of the signs and symptoms but ignoring them. | 10020: “Ignored swelling in neck” |
|  | Misinterpreted signs and symptoms | Participant thought signs and symptoms were related to a different condition/illness. | 10050: “Initially thought the melanoma was an ulcer” |
|  | Not further specified | Participant reports self-delaying but does not expand on the reasons. | 10158: “Self-delaying” |
|  | Personal preference for GP | Participant reports delaying seeking medical attention due to wanting to see a particular GP or having trouble finding a GP they liked/trusted. | 10501: “My usual GP left after 10 years and I found it hard to find another GP that I trusted and liked.” |
|  | Self-medicating | Participant decided to self-medicate before seeking medical attention. | 10231: “…treating with over-the-counter medication.” |
|  | Sought alternative medicine | Participant reports seeking/attending alternative medicine appointments. | 12422: “I had been going to a naturopath previously for three months.” |
|  | Travelling for leisure | Participant travelling for leisure purposes. | 10038: “Travelling abroad” |
|  | Employment commitments | Participant’s work interfered with seeking medical attention. | 14089: “Work is hard to cover…” |
| Healthcare professional | Inattention of healthcare professional | Participant reports not being listened to/ignored by healthcare professional.  Participant reports testing being inadequate. | 10328: “Had been to doctors twice before with same symptoms.” |
|  | Issues with paperwork | Participant reports issues with paperwork/referrals getting lost or not being actioned. | 12171: “Oncologist wanted it done straight away and paperwork got misplaced in Brisbane.” |
|  | Lack of communication from healthcare professional/s | Participant reports not receiving information from healthcare professional/s. | 12141: “Was supposed to have six-month check-ups but fell through the cracks and no-one followed-up.” |
|  | Miscommunication within care team | Participant reports a miscommunication or breakdown of communication within the care team. | 12559: “I was in [rural town] hospital and one doctor wanted to transfer me to [major city], but another doctor said [regional city]. There was a communication breakdown, and it took four days before transferral.” |
|  | Misdiagnosis | Participant reports being misdiagnosed by the healthcare professional. | 10382: “Misdiagnosed as heart burn by GP of 16 years…” |
| Healthcare system | Difficulty obtaining GP appointment | Participant discusses GP not available or long wait time for an appointment. | 12248: “[Regional city] only had two doctors, could not fit in for two weeks when had symptoms…” |
|  | Difficulty obtaining appointment (not further specified) | Participant discusses difficulty obtaining appointment or long wait time but does not specify type of appointment. | 10131: “Took three weeks to get an appointment (booked out).” |
|  | Difficulty obtaining specialist appointment | Participant discusses specialist not available or long wait time for an appointment. | 10183: “I waited six weeks to see a specialist which at the time was not long, but now, since realising diagnosis, it was a while.” |
|  | Difficulty obtaining test appointment | Participant discusses difficulty obtaining appointment or long wait time for a test. | 10141: “It was difficult to get in for a mammogram, put on waiting list.” |
|  | Hospital delay | Participant reports delay in the hospital setting without further details. | 10296: “Delays in hospital.” |
|  | No healthcare service/facility available | Participant reports medical service/facility being unavailable at a location. | 12024: “[Regional city] didn’t have the service.” |
|  | Travel distance | Participant reports needing to travel to healthcare service/facility. | 10018: “Travel to see colon cancer specialist”  12283: “…living remotely…” |
|  | Unable to contact healthcare service/facility | Participant reports being unable to contact healthcare service/facility. | 14198: “Calls from breast screen were 'no caller ID' so couldn't ring them back. They could have left a message with a phone number to ring back. |
| Other | Initial test results negative | Participant reports initial test results were negative. | 12124: “First test was negative, so thought thing were okay. Symptoms didn’t go away so went back to GP”  12638: “One GP did a scan of my bladder which showed nothing.” |
|  | Public holidays |  | 12310: “Delayed going to GP due to Christmas and New Year period” |
| No delay |  | Participant stated no delay or could not recall a delay.  Participant perceived there to be no delay despite explaining a waiting period/delay. | 10164: “It does take a few days to see a GP.  10393: “Two week wait for doctor’s appointment and public holiday delays but par for the course in remote area.” |

GP: General Practitioner
