# Supplementary material for: Rural Cancer Survivors' Perceived Delays in Seeking Medical Attention, Diagnosis and Treatment: Findings From a Large Qualitative Study
Source: Cancer Med. 2025 Jul 21;14(14):e71036. doi: 10.1002/cam4.71036 (PMC12278023; doi:10.1002/cam4.71036)
Supplement: Supplementary file 4 — Table S3. [file CAM4-14-e71036-s007.docx]

**Supplementary Table 3.** Coding framework for responses to the question “Do you feel like the diagnosis was delayed in any way? If yes, why?”

| **Category** | **Code** | **Definition / Rule** | **Example/s** |
| --- | --- | --- | --- |
| Personal | Comorbidity | Participant reports a comorbidity was responsible for the delay such as an illness. | 10116: “Endoscopy which diagnosed cancer was delayed a few days due to comorbidity.” |
|  | Caregiving responsibilities | Participant delayed testing related to their diagnosis or receiving their diagnosis due to caregiving responsibilities to family and friends. | 10310: “I had to take care of my granddaughter…” |
|  | Travelling for leisure | Participant travelling for leisure purposes. | 15102: “Waited one week to receive results because went [on holiday] to [major city].” |
|  | Waiting for preferred testing location | Participant reports waiting to undergo testing at their preferred location. | 15226: “I wanted the colonoscopy and gastroscopy done in [rural town]’s hospital.” |
|  | Not further specified | Participant reports delay on their behalf but does not go on further to explain why. | 10059: “Self-delay two weeks to have biopsy.” |
| Healthcare professional | Inattention of healthcare professional | Participant reports not being listened to by healthcare professional/s.  Reports testing being inadequate. | 10088: “Went back and forward for no reason should have been referred.” |
|  | Issues with paperwork | Participant reports issues with referrals/paperwork. | 12153: “The original referrals went missing between GP and [major city hospital].” |
|  | Lack of communication from healthcare professional/s | Participant reports not receiving or receiving minimal information from healthcare professional/s.  Reports receiving information in a slow manner or required to prompt the healthcare professional for information.  Does not include responses where the participant specifically reports they are waiting for test results, focusses more broadly to information in general or information in relation to testing procedures including referrals. | 10158: “Felt no rush to be given information.”  14203: “Arrived in [rural town] for surgery but told CT scan and biopsy required first - sent to [regional city] for biopsy.” |
|  | Misdiagnosis | Participant reports misdiagnosis. | 10074: “GP originally thought it was sinus.” |
|  | Multiple opinions, scans, or tests required for diagnosis | Participant reports requiring multiple medical opinions and/or tests/scans for diagnosis to be given. | 10130: “Had extensive testing (10-12 biopsy/aspirations/testings).” |
| Healthcare system | Appointment waitlist (not further specified) | Delay perceived due to period participant was required to wait to obtain an appointment but did not specify what for. | 14015: “Took some time to get appointments” |
|  | Finances | Participant reports issues with finances such as affording medical services, private health insurance coverage, and costs to attend appointments. | 10186: “…He was a specialist (urologist) that I could not afford…” |
|  | GP waitlist | Delay perceived due to period participant was required to wait to see a GP. | 12011: “Large delay between initial appointment in June [year]. Made next appointment in October [year] to see GP. |
|  | Hospital waitlist | Delay perceived due to period participant was required to wait due to hospital waitlists (including for surgery), hospital facilities, or the public health system in general. | 12104: “Two months away because ENT doctor was going to do a biopsy, but he could not do it because there were no beds available in ICU.” |
|  | Specialist waitlist | Delay perceived due to period participant was required to wait to see a specialist. | 12426: “Had to wait to see specialist - one month.” |
|  | Test/scan waitlist | Delay perceived due to period participant was required to wait to obtain a test/scan. | 14021: “Took a while to book appointment for a colonoscopy” |
|  | Travel distance | Participant reports needing to travel to healthcare service/facility. | 10018: “Remoteness from specialist practice” |
|  | Waiting for test results | Participant reports delay in receiving test results either due to long reporting times or results not being given to participant (i.e. in a timely manner or lost). | 10492: “My pap smear results came back as abnormal, but I was not notified.” |
| Other | Natural disaster | Participant reports natural disaster or severe weather event caused delay. | 12517: “Cyclone stopped Medivac for two days” |
|  | Negative test results | Participant reports test results were negative. | 12153: “Had tests which came back negative…” |
|  | Public holidays |  | 10363: “Christmas and New Year’s got in the way to do the procedure.” |
|  | Watch and wait period | Participant advised by healthcare professional to watch and wait. | 10008: “Was told by lung cancer team to watch and wait.” |
| Not further specified |  | Participant states there was a delay but did not provide any reasoning. |  |
| No delay |  | Participant stated no delay or could not recall a delay.  Participant perceived there to be no delay despite explaining a waiting period/delay | 12573: “No. The GP told me he could not remove all the cancer and referred me to the dermatologist in [regional city].” |

GP: General Practitioner. ENT: Ear, Nose, and Throat. ICU: Intensive Care Unit. CT: Computed Tomography.
