# Supplementary material for: Rural Cancer Survivors' Perceived Delays in Seeking Medical Attention, Diagnosis and Treatment: Findings From a Large Qualitative Study
Source: Cancer Med. 2025 Jul 21;14(14):e71036. doi: 10.1002/cam4.71036 (PMC12278023; doi:10.1002/cam4.71036)
Supplement: Supplementary file 5 — Table S4. [file CAM4-14-e71036-s005.docx]

**Supplementary Table 4.** Coding framework for responses to the question “Do you feel that starting treatment was delayed in any way? If yes, why?”

| **Category** | **Code** | **Definition / Rule** | **Example/s** |
| --- | --- | --- | --- |
| Personal | Comorbidity | Participant reports a comorbidity was responsible for the delay such as an illness, healing process. | 10001: “Blood pressure issue that needed to be dealt with first.” |
|  | Did not initially meet eligibility criteria | Personal characteristics meant participant was ineligible for treatment/trial. | 10050: “Had to wait until was at Stage 4 to be eligible for clinical trial.” |
|  | Getting affairs in order | Personal matters such as family, personal finances, and getting affairs in order delayed treatment. | 10052: “Had to go back to [rural town] just to get affairs in order” |
|  | Initially declined treatment | Participant reports initially declining the treatment offered. | 10072: “Didn't want chemo[therapy] in the past.” |
|  | Sought further information | Participant sought further information about treatment options before deciding to undertake treatment. | 10160: “I sought more information about treatment and cancer before I made a decision to have the operation and treatment in [major city].” |
|  | Travelling for leisure | Participant travelling for leisure purposes. | 10099: “Could have gone sooner, but had a planned trip [overseas]…” |
|  | Not further specified | Participant reports delay on their behalf but does not go on further to explain why. | 10217: “Personal circumstances.” |
| Healthcare professional | Prolonged treatment planning | Care team delaying treatment whilst determining best treatment plan, including requiring further testing to stage or determine eligibility. | 12076: “Doctors couldn't decide what to do.” |
|  | Issues with paperwork | Participant reports issues with referrals/paperwork. | 10107: “…Hospital did not pass scans onto ENT…” |
|  | Lack of communication from healthcare professional/s | Participant reports that a lack of communication from the healthcare professional/s delayed treatment. | 12154: “No follow up since surgery…” |
|  | Miscommunication within care team | Participant reports miscommunication within the care team. | 12570: “[Regional city hospital] would not start treatment before contact from [major city hospital] – six weeks.” |
|  | Treatment postponed/cancelled by healthcare professional | Treatment was postponed/cancelled by the healthcare professional for reasons such as medical concerns or healthcare professional busy/unavailable. | 10095: “…First plastic surgery date ended up being cancelled due to surgeon pulling out due to concerns of operation…”  10491: “Told needed surgery within 30 days. Next told surgeon was going on holidays for six weeks - date was pushed out again.” |
| Healthcare system | Finances | Participant reports issues with finances including affording medical services, private health insurance coverage, PBS approval, and costs to attend appointments. | 10149: “Could not afford to pay private so was unable to accept two-week later surgery. Had to go on public waitlist…” |
|  | Travel distance | Participant reports needing to travel to healthcare service/facility including accommodation. | 10121: “Coming from [regional city]…” |
|  | Treatment postponed/cancelled due to hospital-related issues | Treatment was postponed/cancelled for reasons related to the hospital or health system in general. | 10185: “Nurse/hospital beds not available so surgery was delayed two weeks.” |
|  | Waiting for appropriate healthcare professional/facilities | Participant reports that delay in treatment was due to needing to wait for the appropriate healthcare professional/facilities to become available. | 10159: “[Regional hospital] did not have appropriate equipment…” |
|  | Waitlist | Participant states waiting for a treatment appointment or public hospital was cause for delay. | 10071: “A few months wait for radiation [therapy].” |
| Other | COVID-19 | Delays related to COVID-19 including isolation and changes in practice. | 12586: “COVID-19 interfered. I was originally referred to [regional city] but then I had a call from [major city hospital] to tell me the specialist doctors were not travelling...” |
|  | More treatment required than first expected | Participant believed treatment was larger/longer than initially expected thus perceived a delay. | 10078: “Surgery treatment was a lot bigger than was expected.” |
|  | Natural disaster | Participant reports natural disaster or weather caused a delay. | 10121: “Cyclone… chose second offered date from surgeon.” |
|  | Public holidays |  | 12189: “Because of all public holidays – could not find surgery times.” |
| Not further specified |  | Participant states there was a delay but did not provide any reasoning. | 10032: “Not happy with treatment process. Treatment should have started a lot earlier.” |
| No delay |  | Participant stated no delay, did not respond to the question, or could not recall a delay.  Participant perceived there to be no delay despite explaining a waiting period/delay | 10344: “No. Placed on 90 day wait list once diagnosed.”  10469: “Not really a delay - considering it was public.” |

PBS = Pharmaceutical Benefits Scheme. ENT = Ear, Nose, and Throat.
Note: If participant described a delay in commencing treatment was due to a delay in diagnosis, they were classified as *No delay* as the question focussed on any reasons for perceived delay in commencing treatment post diagnosis e.g. 10144: “When the professor got diagnosis they said they would call in a couple of hours, for four weeks they did not answer calls and respond.” If participant had not yet commenced treatment and gave no reason for not yet commencing treatment, they were classified as *No delay*.
