# Supplementary material for: Rural Cancer Survivors' Perceived Delays in Seeking Medical Attention, Diagnosis and Treatment: Findings From a Large Qualitative Study
Source: Cancer Med. 2025 Jul 21;14(14):e71036. doi: 10.1002/cam4.71036 (PMC12278023; doi:10.1002/cam4.71036)
Supplement: Supplementary file 6 — Table S5. [file CAM4-14-e71036-s003.docx]

**Supplementary Table 5.** Comparison of participants included in this analysis with those excluded from this analysis due to not completing an interview at baseline, not responding to questions relating to delays in their pathway of initial cancer detection and treatment, or undergoing investigations for cancer but not eventually diagnosed with cancer.

|  | Included in analysis  (n=686) | | Excluded from analysis  (n=125) | | *p-*value^‡^ | 𝜙^§^ / 𝜙_𝑐_^¶^ |
| --- | --- | --- | --- | --- | --- | --- |
|  | n | (%)^†^ | n | (%)^†^ |  |  |
| **Age (years)** |  |  |  |  | 0.8 | 0.046 |
| 20-29 | 4 | (1) | 0 | (0) |  |  |
| 30-39 | 15 | (2) | 2 | (2) |  |  |
| 40-49 | 57 | (8) | 10 | (9) |  |  |
| 50-59 | 127 | (19) | 24 | (22) |  |  |
| 60-69 | 238 | (35) | 33 | (30) |  |  |
| 70+ | 232 | (34) | 42 | (38) |  |  |
| Not reported | 13 |  | 14 |  |  |  |
| **Sex** |  |  |  |  | 0.2 | 0.045 |
| Male | 355 | (53) | 72 | (59) |  |  |
| Female | 318 | (47) | 50 | (41) |  |  |
| Not reported | 13 |  | 3 |  |  |  |
| **Country of birth** |  |  |  |  | **<0.001** | 0.183 |
| Australia | 548 | (80) | 121 | (99) |  |  |
| Other | 138 | (20) | 1 | (1) |  |  |
| Not reported | - |  | 3 |  |  |  |
| **Highest level of education completed** |  |  |  |  | **0.016** | 0.088 |
| High school or lower | 377 | (58) | 75 | (70) |  |  |
| University/ Vocational | 276 | (42) | 32 | (30) |  |  |
| Not reported | 33 |  | 18 |  |  |  |
| **Annual household income**^††^ |  |  |  |  | 0.2 | 0.087 |
| Under $30,000 | 297 | (47) | 47 | (47) |  |  |
| $30,001 to $50,000 | 109 | (17) | 23 | (23) |  |  |
| $50,001 to $80,000 | 79 | (13) | 6 | (6) |  |  |
| $80,001 to $100,000 | 33 | (5) | 4 | (4) |  |  |
| Over $100,001 | 111 | (18) | 21 | (21) |  |  |
| Not reported | 57 |  | 24 |  |  |  |
| **Private health insurance**^‡‡^ |  |  |  |  | 0.4 | 0.050 |
| Yes (fully or partially covered) | 138 | (21) | 0 | (0) |  |  |
| No | 510 | (79) | 6 | (100) |  |  |
| Not reported | 38 |  | 119 |  |  |  |
| **Marital status** |  |  |  |  | 0.3 | 0.071 |
| Single | 97 | (15) | 20 | (19) |  |  |
| In a relationship, de facto, or married | 444 | (68) | 62 | (58) |  |  |
| Divorced | 67 | (10) | 14 | (13) |  |  |
| Widowed | 49 | (7) | 11 | (10) |  |  |
| Not reported | 29 |  | 18 |  |  |  |
| **Geographical remoteness (ARIA)** |  |  |  |  | 0.4 | 0.061 |
| Major city^§§^ | 29 | (4) | 7 | (6) |  |  |
| Inner regional | 307 | (45) | 46 | (37) |  |  |
| Outer regional | 292 | (43) | 61 | (49) |  |  |
| Remote or very remote | 54 | (8) | 11 | (9) |  |  |
| Not reported | 4 |  | - |  |  |  |
| **Area-level disadvantage (SEIFA)** |  |  |  |  | 0.7 | 0.054 |
| Quintile 1 (lowest) | 245 | (36) | 46 | (37) |  |  |
| Quintile 2 | 206 | (30) | 40 | (32) |  |  |
| Quintile 3 | 150 | (22) | 25 | (20) |  |  |
| Quintile 4 | 74 | (11) | 11 | (9) |  |  |
| Quintile 5 (highest) | 7 | (1) | 3 | (2) |  |  |
| Not reported | 4 |  | - |  |  |  |
| **Cancer type** |  |  |  |  | **<0.001** | 0.468 |
| Breast | 125 | (18) | 7 | (6) | **<0.001**^¶¶^ | 0.113 |
| Colorectal | 44 | (6) | 8 | (6) | 0.8^¶¶^ | 0.009 |
| Gynaecological | 61 | (9) | 8 | (6) | 0.5^¶¶^ | 0.022 |
| Head and neck | 105 | (15) | 14 | (11) | 0.4^¶¶^ | 0.029 |
| Lung | 49 | (7) | 6 | (5) | 0.5^¶¶^ | .0.025 |
| Prostate | 82 | (12) | 8 | (6) | 0.1^¶¶^ | 0.054 |
| Skin | 81 | (12) | 11 | (9) | 0.5^¶¶^ | 0.023 |
| Other^†††^ | 130 | (19) | 15 | (12) | 0.1^¶¶^ | 0.051 |
| Unknown primary^‡‡‡^ | 9 | (1) | 36 | (29) | **<0.001**^¶¶^ | 0.462 |
| No diagnosis | - |  | 12 | (10) |  |  |

ARIA: Accessibility/Remoteness Index of Australia. SEIFA: Socio-Economic Indices for Areas. 𝜙: Phi coefficient. 𝜙_𝑐_: Cramér’s V
^†^ Percentage calculated based on non-missing data.
^‡^ *p*-values derived from chi-square tests and Fisher’s exact T-tests.
^§^ Used to measure the effect size where there were two categories (e.g., sex).
^¶^ Used to measure the effect size where there were more than two categories (e.g. age).
^††^ Australian Dollars (2017-2020 depending on date of study recruitment). ^‡‡^ Private health insurance that covered (partially or fully) cancer treatment. ^§§^ Participants classified as living in a major city according to ARIA (23) were included in this sample of rural cancer patients as they had travelled >50 kilometres for cancer care.
^¶¶^ *p*-value adjusted for multiple comparisons using the Bonferroni correction (n=9)
^†††^ Includes anal, bladder, bone, brain, connective tissue/peripheral nerve, eye, gallbladder, kidney, lip, liver, oesophageal, other lymphatic, pancreatic, small intestine, stomach, testicular, thymus, heart, mediastinum and pleura, and thyroid cancers, leukemia, lymphoma, myelodysplastic disease, myeloma, and non-Hodgkins lymphoma.
^‡‡‡^ Participants with an unknown primary cancer site.
