# Supplementary material for: Rural Cancer Survivors' Perceived Delays in Seeking Medical Attention, Diagnosis and Treatment: Findings From a Large Qualitative Study
Source: Cancer Med. 2025 Jul 21;14(14):e71036. doi: 10.1002/cam4.71036 (PMC12278023; doi:10.1002/cam4.71036)
Supplement: Supplementary file 7 — Table S6. [file CAM4-14-e71036-s002.docx]

**Supplementary Table 6.** Comparison of participants who did and did not perceive a delay at three distinct steps in their pathway to initial cancer detection and treatment.

|  | **Seeking medical attention** | | | | | | **Diagnosis** | | | | | | **Commencing** **treatment** | | | | | |
| --- | --- | --- | --- | --- | --- | --- | --- | --- | --- | --- | --- | --- | --- | --- | --- | --- | --- | --- |
|  | Perceived delay (n=132) | | No perceived delay (n=554) | | *p-*value^‡^ | 𝜙^§^ /  𝜙_𝑐_^¶^ | Perceived delay (n=161) | | No perceived delay (n=525) | | *p-*value^‡^ | 𝜙^§^ / 𝜙_𝑐_^¶^ | Perceived delay (n=156) | | No perceived delay (n=530) | | *p-*value^‡^ | 𝜙^§^ / 𝜙_𝑐_^¶^ |
|  | n (%)^†^ | | n (%)^†^ | |  |  | n (%)^†^ | | n (%)^†^ | |  |  | n (%)^†^ | | n (%)^†^ | |  |  |
| **Age (years)** |  |  |  |  | **0.042** | **0.131** |  | |  |  | **0.019** | **0.142** |  |  |  |  | 0.8 | 0.056 |
| 20-29 | 0 (0) | | 4 (1) | | 1.0^††^ | 0.038 | 1 (1) | | 3 (1) | | 1.0^††^ | 0.003 | 0 (0) | | 4 (1) | |  |  |
| 30-39 | 3 (2) | | 12 (2) | | 1.0^††^ | 0.003 | 6 (4) | | 9 (2) | | 0.1^††^ | 0.059 | 2 (1) | | 13 (3) | |  |  |
| 40-49 | 20 (15) | | 37 (7) | | **0.002**^††^ | **0.122** | 21 (13) | | 36 (7) | | 0.01^††^ | 0.096 | 13 (8) | | 44 (8) | |  |  |
| 50-59 | 20 (15) | | 107 (20) | | 0.3^††^ | 0.044 | 27 (32) | | 100 (19) | | 0.5^††^ | 0.025 | 30 (19) | | 97 (19) | |  |  |
| 60-69 | 46 (35) | | 192 (35) | | 1.0^††^ | 0.000 | 62 (39) | | 176 (34) | | 0.2^††^ | 0.045 | 53 (35) | | 185 (35) | |  |  |
| 70+ | 41 (32) | | 191 (35) | | 0.4^††^ | 0.030 | 41 (26) | | 191 (37) | | 0.01^††^ | 0.099 | 55 (35) | | 177 (34) | |  |  |
| Not reported | 2 | | 11 | |  |  | 3 | | 10 | |  |  | 3 | | 10 | |  |  |
| **Sex** |  |  |  |  | 0.2 | 0.045 |  |  |  |  | 0.3 | 0.041 |  |  |  |  | 0.07 | 0.069 |
| Male | 62 (48) | | 293 (54) | |  |  | 77 (49) | | 278 (54) | |  |  | 71 (47) | | 284 (55) | |  |  |
| Female | 67 (52) | | 251 (46) | |  |  | 80 (51) | | 238 (46) | |  |  | 82 (53) | | 236 (45) | |  |  |
| Not reported | 3 | | 10 | |  |  | 4 | | 9 | |  |  | 3 | | 10 | |  |  |
| **Country of birth** |  |  |  |  | 0.7 | 0.014 |  |  |  |  | 0.4 | 0.029 |  |  |  |  | 0.7 | 0.014 |
| Australia | 107 (81) | | 441 (80) | |  |  | 132 (82) | | 416 (79) | |  |  | 123 (79) | | 425 (80) | |  |  |
| Other | 25 (19) | | 113 (20) | |  |  | 29 (18) | | 109 (21) | |  |  | 33 (21) | | 105 (20) | |  |  |
| **Highest level of education completed** |  |  |  |  | 0.6 | 0.022 |  |  |  |  | **0.042** | **0.080** |  |  |  |  | 0.1 | 0.070 |
| High school or lower | 70 (56) | | 307 (58) | |  |  | 78 (51) | | 299 (60) | |  |  | 76 (51) | | 301 (60) | |  |  |
| University / Vocational | 56 (44) | | 220 (42) | |  |  | 76 (49) | | 200 (40) | |  |  | 72 (49) | | 204 (40) | |  |  |
| Not reported | 6 | | 27 | |  |  | 7 | | 26 | |  |  | 8 | | 25 | |  |  |
| **Annual household income**^‡‡^ |  |  |  |  | 0.4 | 0.077 |  |  |  |  | 0.8 | 0.054 |  |  |  |  | 0.2 | 0.095 |
| Under $30,000 | 54 (44) | | 243 (48) | |  |  | 65 (42) | | 232 (49) | |  |  | 66 (46) | | 231 (48) | |  |  |
| $30,001 to $50,000 | 24 (20) | | 85 (17) | |  |  | 29 (19) | | 80 (17) | |  |  | 29 (20) | | 80 (16) | |  |  |
| $50,001 to $80,000 | 14 (11) | | 65 (13) | |  |  | 21 (14) | | 58 (12) | |  |  | 12 (8) | | 67 (14) | |  |  |
| $80,001 to $100,000 | 4 (3) | | 29 (6) | |  |  | 9 (6) | | 24 (5) | |  |  | 11 (8) | | 22 (5) | |  |  |
| Over $100,001 | 27 (22) | | 84 (17) | |  |  | 29 (19) | | 82 (16) | |  |  | 25 (17) | | 86 (18) | |  |  |
| Not reported | 9 | | 48 | |  |  | 8 | | 49 | |  |  | 13 | | 44 | |  |  |
| **Private health insurance**^§§^ |  |  |  |  | 0.4 | 0.033 |  |  |  |  | 0.2 | 0.048 |  |  |  |  | 0.5 | 0.026 |
| Yes (full or partial coverage) | 23 (19) | | 115 (22) | |  |  | 38 (25) | | 100 (20) | |  |  | 34 (23) | | 104 (21) | |  |  |
| No | 101 (81) | | 409 (78) | |  |  | 115 (75) | | 395 (80) | |  |  | 112 (77) | | 398 (79) | |  |  |
| Not reported | 8 | | 30 | |  |  | 8 | | 30 | |  |  | 10 | | 28 | |  |  |
| **Marital status** |  |  |  |  | 0.7 | 0.046 |  |  |  |  | 0.3 | 0.078 |  |  |  |  | 0.8 | 0.035 |
| Single | 17 (14) | | 80 (15) | |  |  | 23 (15) | | 45 (9) | |  |  | 22 (15) | | 75 (15) | |  |  |
| In a relationship, de facto, or married | 86 (69) | | 358 (67) | |  |  | 102 (65) | | 342 (68) | |  |  | 103 (68) | | 341 (67) | |  |  |
| Divorced | 15 (12) | | 52 (10) | |  |  | 22 (14) | | 74 (15) | |  |  | 17 (11) | | 50 (10) | |  |  |
| Widowed | 7 (6) | | 42 (8) | |  |  | 9 (6) | | 40 (8) | |  |  | 9 (6) | | 40 (8) | |  |  |
| Not reported | 7 | | 22 | |  |  | 5 | | 24 | |  |  | 5 | | 24 | |  |  |
| **Geographical remoteness (ARIA)** |  |  |  |  | 0.6 | 0.050 |  |  |  |  | **0.048** | **0.108** |  |  |  |  | 0.3 | 0.077 |
| Major city^¶¶^ | 5 (4) | | 24 (4) | |  |  | 12 (7) | | 17 (3) | | 0.4^†††^ | 0.056 | 9 (6) | | 20 (4) | |  |  |
| Inner regional | 61 (46) | | 246 (45) | |  |  | 78 (48) | | 229 (44) | | 1.0^†††^ | 0.002 | 78 (50) | | 229 (44) | |  |  |
| Outer regional | 59 (45) | | 233 (42) | |  |  | 58 (36) | | 234 (45) | | 0.8^†††^ | 0.019 | 57 (37) | | 235 (45) | |  |  |
| Remote or very remote | 7 (5) | | 47 (9) | |  |  | 13 (8) | | 41 (8) | | 0.8^†††^ | 0.015 | 12 (8) | | 42 (8) | |  |  |
| Not reported | - | | 4 | |  |  | - | | 4 | |  |  | - | | 4 | |  |  |
| **Area-level disadvantage (SEIFA)** |  |  |  |  | 0.9 | 0.040 |  |  |  |  | 0.8 | 0.047 |  |  |  |  | 0.1 | 0.106 |
| Quintile 1 (lowest) | 43 (33) | | 202 (37) | |  |  | 57 (35) | | 188 (36) | |  |  | 58 (37) | | 187 (36) | |  |  |
| Quintile 2 | 42 (32) | | 164 (30) | |  |  | 44 (27) | | 162 (31) | |  |  | 49 (32) | | 157 (30) | |  |  |
| Quintile 3 | 30 (23) | | 120 (22) | |  |  | 40 (25) | | 110 (21) | |  |  | 26 (17) | | 124 (24) | |  |  |
| Quintile 4 | 15 (11) | | 59 (11) | |  |  | 18 (11) | | 56 (11) | |  |  | 23 (15) | | 51 (10) | |  |  |
| Quintile 5 (highest) | 2 (2) | | 5 (1) | |  |  | 2 (1) | | 5 (1) | |  |  | 0 (0) | | 7 (1) | |  |  |
| Not reported | - | | 4 | |  |  | - | | 4 | |  |  | - | | 4 | |  |  |
| **Cancer type** |  |  |  |  | **<0.001** | **0.202** |  |  |  |  | 0.1 | 0.141 |  |  |  |  | 0.3 | 0.119 |
| Breast | 18 (14) | | 107 (19) | | 0.1^§§§^ | 0.058 | 21 (13) | | 104 (20) | |  |  | 27 (17) | | 98 (19) | |  |  |
| Colorectal | 8 (6) | | 36 (6) | | 0.9^§§§^ | 0.007 | 11 (7) | | 33 (6) | |  |  | 9 (6) | | 35 (7) | |  |  |
| Gynaecological | 22 (17) | | 39 (7) | | **<0.001**^§§§^ | **0.133** | 22 (14) | | 39 (7) | |  |  | 20 (13) | | 41 (8) | |  |  |
| Head and neck | 22 (17) | | 83 (15) | | 0.629^§§§^ | 0.018 | 23 (14) | | 82 (16) | |  |  | 16 (10) | | 89 (17) | |  |  |
| Lung | 10 (8) | | 39 (7) | | 0.8^§§§^ | 0.008 | 10 (6) | | 39 (7) | |  |  | 11 (7) | | 38 (7) | |  |  |
| Prostate | 7 (5) | | 75 (14) | | 0.009^§§§^ | 0.100 | 14 (9) | | 68 (13) | |  |  | 22 (14) | | 60 (11) | |  |  |
| Skin | 18 (14) | | 63 (11) | | 0.5^§§§^ | 0.028 | 19 (12) | | 62 (12) | |  |  | 15 (10) | | 66 (12) | |  |  |
| Other^¶¶¶^ | 22 (17) | | 108 (19) | | 0.5^§§§^ | 0.028 | 38 (24) | | 92 (18) | |  |  | 33 (21) | | 97 (18) | |  |  |
| Unknown primary^††††^ | 5 (4) | | 4 (1) | | **0.005**^§§§^ | **0.106** | 3 (2) | | 6 (1) | |  |  | 3 (2) | | 6 (1) | |  |  |

ARIA: Accessibility/Remoteness Index of Australia. SEIFA: Socio-Economic Indices for Areas. 𝜙: Phi coefficient. 𝜙_𝑐_: Cramér’s V
^†^ Percentage calculated based on non-missing data.
^‡^ *p*-values derived from chi-square tests and Fisher’s exact T-tests.
^§^ Used to measure the effect size where there were two categories (e.g., sex).
^¶^ Used to measure the effect size where there were more than two categories (e.g. age).
^††^ *p*-value adjusted for multiple comparisons using the Bonferroni correction (n=6)
^‡‡^ Australian Dollars (2017-2020 depending on date of study recruitment). ^§§^ Private health insurance that covered (partially or fully) cancer treatment. ^¶¶^ Participants classified as living in a major city according to ARIA (23) were included in this sample of rural cancer patients as they had travelled >50 kilometres for cancer care.
^†††^ *p*-value adjusted for multiple comparisons using the Bonferroni correction (n=4)
^§§§^ *p*-value adjusted for multiple comparisons using the Bonferroni correction (n=9)
^¶¶¶^ Includes anal, bladder, bone, brain, connective tissue/peripheral nerve, eye, gallbladder, kidney, lip, liver, oesophageal, other lymphatic, pancreatic, small intestine, stomach, testicular, thymus, heart, mediastinum and pleura, and thyroid cancers, leukemia, lymphoma, myelodysplastic disease, myeloma, and non-Hodgkins lymphoma.
^††††^ Participants with an unknown primary cancer site.
